# “It's normal to be afraid”: attacks on healthcare in Ouaka, Haute-Kotto, and Vakaga prefectures of the Central African Republic, 2016–2020

**DOI:** 10.1186/s13031-024-00610-8

**Published:** 2024-08-27

**Authors:** Natalya Kostandova, Jennifer OKeeffe, Blaise Bienvenu Ali, Pierre Somsé, Audrey Mahieu, Odilon Guesset Bingou, Sebastien Dackpa, Gerard Mbonimpa, Leonard Rubenstein

**Affiliations:** 1 Johns Hopkins Bloomberg School of Public Health, Baltimore, MD USA; 2 Institut Centrafricain des Statistiques et des Etudes Economiques et Sociales, Bangui, Central African Republic; 3 Ministère de la Santé et de la Population, Bangui, Central African Republic; 4 Geneva Centre of Humanitarian Studies, University of Geneva, Geneva, Switzerland; 5 International Medical Corps, Bangui, Central African Republic

**Keywords:** Central African Republic, Armed conflicts, Health personnel, Health facilities, Health workforce, Health services accessibility, Violence, Public health, Human rights abuses, Attacks on healthcare

## Abstract

**Introduction:**

Attacks on healthcare have further weakened the already fragile health system in the Central African Republic. We investigated attacks on healthcare in three conflict-affected prefectures—Ouaka, Haute-Kotto, and Vakaga—from 2016 to 2020. The study aim was to gain an in-depth understanding of the immediate and long-term effects of attacks on healthcare workers, facilities, supply chain, quality of care, and other components of the health system. We provide a qualitative description of the incidents, assess their impacts, identify mitigation efforts, and discuss challenges to recovery.

**Methods:**

We used purposive and snowball sampling to identify participants in the study. Semi-structured key informant interviews were conducted with administrative and health authorities, front-line personnel, and staff of non-governmental organizations. Interviews were done in Sango, French, or English. Recorded interviews were transcribed and notes taken for non-recorded interviews. Transcripts and notes were analyzed using inductive coding, allowing participant responses to guide findings.

**Results:**

Of 126 attacks identified over the study period, 36 key informants discussed 39 attacks. Attacks included killings, physical and sexual assault, abductions, arson, shelling with grenades, pillage, occupations, and verbal threats. The violence led to extended closures and debilitating shortages in healthcare services, disproportionately affecting vulnerable populations, such as children under five, or people who are elderly, chronically ill, or displaced. Healthcare workers faced psychological trauma and moral injury from repeated attacks and the inability to provide adequate care. Personnel and communities made enormous efforts to mitigate impacts, and advocate for assistance. They were limited by failed reporting mechanisms, ongoing insecurity, persistent lack of resources and external support.

**Conclusion:**

Effective strategies to safeguard healthcare from violence exist but better support for communities and health workers is essential, including measures to assess needs, enhance security, and facilitate recovery by quickly rebuilding, resupplying, and re-staffing facilities. CAR’s government, international organizations, and donors should make concerted efforts to improve reporting mechanisms and end impunity for perpetrators. Their investment in community organizations and long-term health system support, especially for health worker training, salaries, and psychosocial care, are vital steps towards building resilience against and mitigating the impacts of attacks on healthcare.

**Supplementary Information:**

The online version contains supplementary material available at 10.1186/s13031-024-00610-8.

## Introduction

The Central African Republic (CAR), one of the poorest countries in the world, has been suffering a brutal internal conflict for more than a decade. A variety of non-state armed groups control most of the country, intimidating and inflicting violence on local populations. Assessments have estimated that ten of the country’s 20 prefectures, comprising 47.5% of the population, were partially or entirely controlled by rebel groups as of July 2021 [1]. The country ranks 191 out of 193 countries on the UN Human Development Index [2]. Both maternal and infant mortality rank as the 5th worst globally and malaria and malnutrition are highly prevalent [3]. More than a million people are displaced and half of its population of 6.1 million people require humanitarian assistance [4]. People living in displacement sites often lack access to basic needs, including food, clean water, and sanitation [3].

The health system was weak before the conflict and has since been even further weakened due to damage experienced during the conflict. Government spending on health was just over $3.50 per person per year in 2023 [5]. Only half of the health facilities are fully functional, and there is a severe shortage of trained personnel, with a ratio of only 0.6 doctors for every 100,000 people, one of the lowest in the world [6]. More than 280 local, international, and UN organizations operate in the country providing aid to the population and support to the national health system, including providing staff and support for state-run health facilities [7]. While these efforts are crucial to save lives in the short-term, they cannot address the health needs of the entire population or substitute for a sustainable health system, particularly in the conflict-affected regions of the country where health needs are greatest.

The health system has been further burdened by violent attacks on and threats to health facilities and personnel during the conflict. Although reporting of attacks is limited and irregular, the Safeguarding Health in Conflict Coalition reported 52 attacks against healthcare in 2017 and 47 in 2018 [8]. The impact of violence against health care in conflict has gained increasing attention in the last decade, but except for attention to mental health impacts, peer-reviewed studies have mainly been conducted in middle-income countries [9, 10], or low-income, conflict-affected countries in the Middle East [11–13]. In low-income, conflict-affected countries in sub-Saharan Africa, where health care services were often weak before the conflict, with few exceptions [14], information on attacks is limited to data collected during brief surveys, such as those conducted in South Sudan [15] and Nigeria [16], and qualitative studies of family, and community and patient violence in the Democratic Republic of Congo [17].

**Fig. 1:**
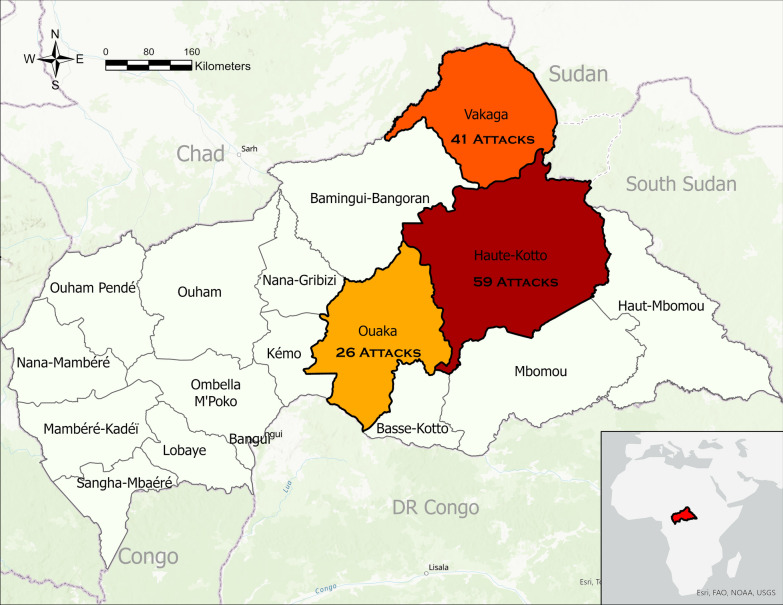
Attacks on Health Care in Haute-Kotto, Ouaka, and Vakaga Prefectures, CAR, 2016–2020, identified using primary data collection and secondary data sources

Three conflict-affected prefectures were considered in the study: Haute-Kotto, Ouaka, and Vakaga. They were supported by the non-governmental organization (NGO) the International Medical Corps (IMC) and were chosen because IMC was able to facilitate contact with participants, access to related data, and assist in triangulation of findings. From 2016 through 2020, there was a heavy presence and activity of armed groups in these three prefectures (Fig. 1). Populations in Haute-Kotto, Ouaka, and Vakaga were repeatedly exposed to severe violence and forced displacement. While many humanitarian actors were present in the prefectures, their activities concentrated in and around the capitals of Bambari, Birao and Bria. Large parts of the prefectures and their capitals were engaged in active conflict. To understand the effects of attacks on healthcare on patients, communities, and providers in the three prefectures, we conducted a mixed methods study using key informant interviews and interrupted time series analysis. The study objectives were to assess the immediate and long-term impacts of the attacks, identify community and partner-led mitigation efforts, and describe the substantial challenges faced by communities, providers, and other actors in restoring access to and quality of healthcare services. Here, we present the qualitative component of the study.

## Methods

### Study design

We employed qualitative methods using key informant interviews to analyze the impact of attacks on healthcare. The study was an initiative of the *Researching the Impact of Attacks on Healthcare Consortium* [18] conducted by Johns Hopkins University’s Bloomberg School of Public Health, the Ministry of Health and Population (MSP), the Central African Institute for Statistics and Economic and Social Studies (ICASEES), and the Geneva Centre of Humanitarian Studies at the University of Geneva.

### Study setting and period

All attacks on healthcare in the conflict-affected prefectures of Haute-Kotto, Ouaka, and Vakaga in the Central African Republic from January 2016 to December 2020 were considered in the study. Initially, the study limited inclusion to participants who knew of attacks in IMC zones of responsibility. However, during interviews, participants identified attacks outside IMC supported areas in the three prefectures, which were also included for their relevance to study objectives. While there were widespread attacks against the general population or other institutions in the three prefectures, this information was excluded from analysis as it fell outside the scope of the study aims to investigate the impact of attacks on healthcare in CAR.

### Sampling and participants

We used key informants from IMC and the MSP to identify an initial list of participants in the study, comprised of administrative authorities, district and regional health authorities, health personnel working in MSP facilities supported by IMC, and IMC program personnel. Initial participants were selected based on previous knowledge of individual attacks that occurred in the study area and during the study period. We then used snowball sampling to identify further participants who may have information about specific attacks due to their professional positions. An attack on healthcare was defined for participants as “Acts of threat, coercion or physical or verbal violence, which hinder the provision of health care. These attacks can target healthcare personnel on duty, patients, health facilities or medical transports”. All participants who had knowledge of a specific attack on healthcare over the study period and provided informed consent were included in the study. We excluded participants who had knowledge of attacks solely outside the study period or where healthcare was not directly targeted. Participants consisted of CAR administrative authorities, MSP front-line health personnel, regional and district health directors, and staff from NGOs.

### Data collection

Interviewers and transcribers from ICASEES participated in an in-person three-day research training that covered the informed consent process, psychological first aid, research ethics, interviewer skills, contents of the interview guide, and pilot interviews. Interviewers conducted semi-structured key informant interviews (KIIs) with all participants using a pre-developed guide (*Supplementary materials)*. We explained the purpose of the study to all prospective participants and described their right to withdraw from the study at any time. Additionally, we noted that no identifying information would appear in the transcripts, summaries, or manuscript, with personal identifiers limited to voice in interviews.

The majority of KIIs were conducted by researchers at ICASEES, with the remainder conducted by other members of the research team (NK + AM). Interviews were conducted in the language of the participant’s choice, Sango, French, or English. All recorded interviews were transcribed into French for analysis. For participants who declined recording, notes were taken in French or English during the interviews. Interviews were carried out remotely by phone or using encrypted calling software (Skype, Zoom). Interview recordings were deleted after transcription was completed. Transcriptions and notes were stored in password-protected encrypted cloud-based folders only accessible to the study team.

Data collection occurred in three phases: May–June 2022, December 2022-January 2023, and June–August 2023. Initial delays stemmed from operational adjustments and additional training. In the final phase, the team ensured thorough coverage by interviewing participants who they had not been able to reach to achieve saturation.

### Data analysis

All transcribed interviews and notes were analyzed in English with Nvivo software version 14.23.3 (61) using an inductive coding approach [19]. Inductive coding allowed participant responses to guide the development of themes, providing a data-driven categorization of findings. An initial, unrestricted line-by-line coding was conducted on a subset of four transcripts to develop preliminary codes (JO). These were then edited, revised, added to, or deleted to reflect the data in subsequent transcripts. The iterative process ensured that the final set of themes accurately represented the data. To increase rigor in the dependability and confirmability of findings, a subset of 4 transcripts (> 10%) was recoded by a second member of the research team (NK). Any differences were arbitrated, and a consensus reached amongst the research team.

The saliency of themes was determined both by the frequency of mention across different interviews as well as the depth of description by the participant. Frequency of mention within individual transcripts was not considered in determining the significance of themes. The dual emphasis on frequency across and depth within transcripts provided a mechanism for identifying the most important themes to participants, ensuring findings were grounded in their experiences.

To complement the thematic analysis, we created a comprehensive data matrix. This matrix served to extract and organize key information from each transcript, providing a structured overview of the data. The matrix included variables such as the place and date of the attack, the position of the participant within the healthcare system, a detailed description of the attack, its immediate impact, and the mid- to long-term effects. Additionally, the matrix captured the effects of the attacks on neighboring health facilities, including absorbing patients from facilities that had been attacked, and external factors that may have exacerbated the impact of attacks like challenges in the supply chain, which hindered the ability to resupply the facility after it was pillaged.

### IRB approval

The study was approved by Comité Ethique et Scientifique at the University of Bangui, Central African Republic (N°27/UB/FACSS/IPC/CES/021), by the Comission d’éthique Universitaire at the University of Geneva (CUREG.202101.11), and by the Institutional Review Board Office at Johns Hopkins University (Determination notice FWA #00000287). The study received Quitus statistique from the Comité Technique de Programmation des Activité Statistiques (CTPAS), Central African Republic (N°0377/2022/MEPC/DIRCAB/ICASEES/DMNER).

## Results

### Interviews

In total, 126 attacks over the study area and period were identified. From these attacks, we interviewed 41 participants, of which 36 met inclusion criteria. Five male participants had knowledge solely of attacks outside of the study period, three heads of health facilities, and two NGO staff. The included participants comprised 34 men and 2 women, and included staff of health facilities, NGO staff, district and regional health authorities, administrative authorities, and members of the community health board, a local governance structure that provides oversight to the health facility and supports health initiatives in the community (Table 1).

**Table 1:** Profile of participants in key informant interviews

Participant profile	Number of participants
Head of health structure	14
Staff of non-governmental organizations	8
District and regional health authorities	5
Health structure personnel (excluding heads of health facilities)	4
Administrative authorities	3
Members of the community health board	2

Interviews ranged from 35 to 80 min in duration (excluding time for informed consent). In the included interviews, participants described 39 different attacks: 16 in Vakaga, 12 in Haute-Kotto, and 11 in Ouaka. The timeframe of these attacks ranged from the earliest in February 2016 to the latest in December 2020. Of the 39 attacks, 12 occurred at hospitals, 12 at health centers, 10 at health posts, and 5 on the road to or from a health facility.

### Contextual overview

Non-state armed groups, including local militias and those originating in Sudan and Chad, attacked health care in the three study prefectures in the context of ongoing violence and insecurity that led to population displacement, food insecurity, poor water and sanitation, infectious disease outbreaks, financial barriers to access to health care, human resources shortages, and distrust of the health system. Insecurity also rendered travel on roads dangerous, complicating both access to care and medical supply to facilities. The population suffered enormously. One participant said:

The extent of the damage.... I don't know how to describe it, but when there are attacks and destruction like that you can imagine the impact on the community. The community is already a Central African who has no means to take care of himself. Someone comes and destroys everything, destroys his house, burns everything, everything he finds, everything he has.... He becomes a beggar, what can he do if there aren't people of good faith to support him… He will die of malnutrition.

Attacks on health care were so ubiquitous that many participants had trouble differentiating attacks that occurred during the study period with attacks that happened before and after. Some participants wanted to convey the size and scale of the conflict while others struggled to differentiate the events and impacts of various attacks.

### Attacks on facilities

Participants reported that attacks on health facilities and personnel occurred both as one facet of broader assaults on villages or towns and on roads as well as in instances where violence specifically targeted a health facility. Staff at facilities sometimes received a small advance warning that an attack was imminent from people living in neighboring villages or travelers passing through the area. More often, though, participants reported that an attack came without notice, forcing healthcare workers, along with the local population, to be subjected to violence or to flee with little food, clothing, or household supplies. Some lived in fields and bush for weeks or months depending on the position of assailants and the security context.

Staff showed remarkable bravery and judgment in many instances. In one case, in the absence of their own form of transport, staff rented a motorcycle to evacuate the maternity and the in-patient wards before the attackers arrived. Another participant shared the story of a facility midwife who had the foresight to instruct others to carry boxes of medications with them while they fled the facility. These were later used to care for the population while waiting for the facility to be restocked.

As reported by participants, the perceived reasons for the attacks varied: (1) To loot the facility of money, medications, and goods the attackers could sell or use; (2) to sow fear in the community; (3) to retaliate for allegedly inadequate care; (4) to gain priority care for members of the group; and (5) in retribution against healthcare personnel for providing care to the group’s enemies. Armed groups sometimes made intentional efforts to cause as much damage as possible, using grenades, guns, and setting fire to the structure, vehicles, and materials. One participant described an incident where a battle had taken place near the town,

A fighter was seriously wounded on the battlefield when his colleagues were taken to the facility. A doctor was operating [on another patient] in the operating theatre, and his colleagues burst into the facility demanding that he be given quality care, otherwise the doctor would be held responsible if he died.

Pillaging could be extensive, sometimes including complete destruction of the facility. Participants reported that an NGO-operated health facility was likely more vulnerable because it had greater resources and could generate more profits for attackers. One respondent explained:

What happened in these health facilities in the first place, people think that there is money in the hospital, they come first to look for the cash box and search for the money. If they do not find the money, they take medicines to resell. In the opposite case, if there is no medicine, they take materials to resell. In the worst cases, if they don't find something that can allow them to have some money to survive, they burn what is there.

Often after pillaging, combatants destroyed any materials and medications that they were either uninterested in or unable to carry with them. In some cases, pillaging was accompanied by arson, launching explosives, such as grenades, or otherwise damaging the facility. Health facilities were also subjected to takeover by armed groups, either to use it as a military base, strategic positioning, or to demand care for their sick and wounded. In one case, a participant said:

The center was occupied by the armed men, and it was their base. It was from there that they committed [assaults] on the village.

Health facilities were also affected when people displaced by an attack sought refuge in them. Sheltering internally displaced persons (IDPs) disrupted services and sometimes heightened tensions and the risk of attacks. One participant said.

The populations of village [X] and village [Y] took refuge in the hospital, where they stayed for two to three years…The living conditions were very difficult. They had nothing to eat, they were not taken care of. They benefitted from some care after several months of inactivity. Some died because of the lack of care. It was total disorder. They did not know how to survive.

Repeat attacks on the same health facility disrupted recovery and further weakened the health system. Attacks in neighboring areas had repercussions for facilities nearby.

These attacks were part of a series of repeated and organized attacks themselves, so they are not sporadic or targeted attacks.

### Attacks on healthcare workers, staff and patients, and families

Participants reported that healthcare workers often faced high risks of attack due to the nature of their jobs. As demonstrated above, they worked with resources such as medicines, forms of transport, liquid cash, and other materials that could attract the attention of armed groups. They sometimes worked in structures that had the best construction in the area. As healthcare workers were often obliged to travel for their work, resupply their stock, submit reports, and conduct outreach activities, they were vulnerable to attacks on roads. Despite this, healthcare workers were obliged to collaborate, cooperate and, above all, treat combatants and attackers, along with the civilian population, without distinction.

Participants reported that armed groups killed, raped, abducted, beat, and threatened healthcare workers and their families. Sometimes they were attacked during healthcare activities or during the pillaging of their facility. In other cases, they were specifically targeted for the same reasons as facilities, e.g., dissatisfaction with quality of care, providing care to enemies, sowing fear, and to destabilize the area. They were also attacked because they were seen as wealthy or for resisting demands from an armed group.

While sometimes health personnel were killed without planning during the chaos of pillaging, often participants perceived that killings were intentional. The murders were specifically aimed at the personnel as acts of retribution, while traveling to restock supplies or while visiting sites for care or vaccinations.

When one of the members of the armed group died in the hospital, they blamed the health workers directly. They killed the head of the hospital, burst into the hospital as they pleased with grenades, knives and weapons and threatened the [other] health workers.

Some staff were beaten or wounded and died later of their injuries. Several participants reported that colleagues were killed in their homes. One participant described how a colleague had survived an attack but was so badly beaten that they continued to suffer from their injuries at the time of the interview, more than two years after the attack had occurred. A participant explained:

Unfortunately, he came across armed groups who beat him up and he suffered many injuries. To this day, he continues to suffer the after-effects of this attack. He is still not well.

Some abductions were brief, lasting several hours, while the armed groups sought ransom. Others lasted months or years. One participant described the kidnapping of five staff in 2019. Two of the five remained missing, with no word of them at the time of interview in December 2022.

They burst into the [facility] sequestering a man and four women... A few moments later, they released the two older women who told us that the man who was sequestered had been badly beaten... The other two sequestered women have not yet been released and [the four women] had been raped...

Healthcare workers were also frequently threatened with violence. One participant explained:

Either it is verbal threats that meet their [demands] or corporal punishment and even that can end in death. I personally have been the victim of a verbal threat but not corporal punishment yet. I [prefer to be] compliant.

Threats often were made against personnel and patients when an armed group set up camp or entered a town or village. Verbal threats were used to coerce healthcare personnel into hiring members of armed groups for health activities, such as vaccination campaigns or to obtain priority care at facilities. A participant also recalled a situation when a gunshot survivor was brought to a hospital with an ambulance, underwent surgery, and was threatened by leaders of another armed group and caretakers of other patients. While health personnel were able to move the patient to another hospital room to separate them from patients from another armed group, and remained vigilant to ensure the patient was not attacked, the patient left the hospital well before they should have, because the hospital could not assure their safety.

Family members of healthcare workers were also at risk. In one attack, the head of the health center was kidnapped, tied up, and beaten to death. A family member of the health center staff was likewise kidnapped and murdered, presumably due to their association with the health center staff.

Armed groups also killed patients. One respondent said:

They broke into the health center, shot everywhere, even at the sick, even those who were on serums…The displaced, the sick on the hospital beds were killed by the armed groups.

As with the population, healthcare personnel suffered loss of their homes, transportation, and personal items in the attacks, and were displaced or returned to their original homes. One said:

[After the attack], I lived at [a site for internally displaced people] for 3 years. I returned home [this year]. I now sleep in a tarpaulin.

### Impacts of attacks

#### Impacts on facilities and communities

Participants reported that attacks on health faculties interrupted services for varying periods of time. In several cases, certain health services were closed permanently. Participants reported that the population was often left to look for alternate sources of care, either traveling great distances to reach neighboring facilities or turning to traditional sources of care. One participant stated that the entire village, including healthcare personnel and volunteers, had fled to Sudan.

Great efforts were made to reopen services soon after an attack, but staff and communities faced significant administrative and operational challenges. Health center staff struggled to repair the facility and rid it of debris. The direct effects of attacks were often exacerbated by general insecurity in the region, which made it difficult to transport construction materials and resupply the facilities. Some participants reported that they needed approval from senior health officials to reopen services. Reluctance to reopen in some cases was tied to the risks of subsequent attacks.

When facilities reopened despite damage from an attack, which could happen in a matter of weeks for the more accessible facilities or years for the more inaccessible ones, they faced enormous challenges. Damage to the structure could inhibit personnel from ensuring proper triage, patient flow, or even basic shelter from the elements for patients. Participants spoke of roofs so damaged that they were torn off completely.

In addition to structural damage, staff were also often unable to maintain quality of care because of lack of supplies, equipment, and inadequately trained staff. Sometimes, a re-opened facility would operate with temporarily reduced services or permanently discontinue certain services. Discontinued services included those intended for the most vulnerable groups: maternity and reproductive health, treatment of acute malnutrition, surgery, and HIV care. People being treated for TB and HIV often missed treatments, potentially increasing drug resistance. Following one attack, a facility suspended vaccination outreach, providing vaccines only on-site, which created an additional barrier to access. Some facilities never fully recovered, operating with more limited resources for months or years. In the worst cases, facilities closed permanently, leaving communities without care and overburdening neighboring facilities.

Participants described the physical and mental health impacts of attacks, displacement and restrictions of movement on patients and their families. These often extended for long periods and were exacerbated by community destabilization and sometimes dissolution. After attacks, communities were more vulnerable to death and disease, both as direct effects of the attack, due to displacement, death, or injury, or as an indirect effect through psychological trauma, the deterioration of social networks, inability to work, and inferior living conditions. Damage to the health system meant that these health concerns were less likely to be diagnosed or treated in early stages, or even properly treated at all, leading to worse outcomes. The most vulnerable were elderly people, young children, and people living with disabilities.

One participant said:

At the moment, there is no health facility in [the town] and the population is suffering. Children are not vaccinated, there is a risk of epidemics and pregnant women often die as a result of unattended births at home.

Utilization of health facilities often changed even after reopening. In some cases, there were few cases reporting to facilities in the period immediately following an attack, as people were afraid to travel to seek healthcare, even for short distances. One participant said:

First of all, when even [healthy] people can't get around, sick people can't get around, even anyone can't get around, just the belligerents. That's one [part] and then there was also the [general] problem of accessibility.

In some cases, after the initial decline in utilization, there was an upsurge as the situation stabilized and travel became easier. In one community, in the period after the attack, people declined to be hospitalized or refused to stay overnight out of fear for their safety. People also relied more on traditional medicine to treat the sick and injured, either because of the risk of traveling, fear of health seeking, or because they knew that the health center was non-functional or under-equipped after an attack. Another participant explained,

After these events, they were too scared to bring their children to the hospital. Those that lived far preferred to treat their children with traditional medicines, which meant that there were a lot of deaths.

Attacks on health facilities also contributed to displacement of communities. Participants perceived displacement as strongly linked to poorer health outcomes from inadequate shelter, deficient WASH conditions, food insecurity, and increasing susceptibility to malaria (due to sleeping outside and lack of bed nets) and other parasitic diseases. One participant said:

[People] first fled the fighting at their home to come [to this village], and the same fate followed them here again, so they decided to flee to go elsewhere… They had fled the fighting, and they had to move to yet another destination after the attack on our health structure.

Participants reported an increase in mortality, in part because of delays in seeking health care. Often, individuals waited so long to bring a sick family or friend to the health structure that the individual was no longer treatable. In these instances, personnel could do nothing more than make people comfortable as they died. One said,

The inpatient mortality^1^…. when people arrive at a late stage of sickness, and we the healthcare personnel can’t do anything, it ends with death…but people count on this increase in mortality as normal. It’s tied to the effects of the insecurity and all [its] corollaries.

Inpatient mortality here refers to all deaths at the health facilities. Participants also described the psychological effects of generalized and repeated attacks on civilians, including the murder of family members and friends, beatings, rape, attempted killings, and other acts that had been perpetrated against them by armed groups. Many community members were in a state of shock after an attack, experiencing feelings of fear, anxiety, anger, depression, and detachment.

There are too many consequences and difficulties. People are always on their toes. Fear wins everyone over. You can't buy anything of value to keep. It plays a big part in the population. People are afraid. I, for example, don't want to have children, because if I do, how are you going to run away? I [would be] obliged to make space for a child, so that when [attacks] happen, he can run and walk by himself. Because you may have to run at any time. The consequences are numerous…

#### Impacts on healthcare workers

Like other community members, healthcare workers suffered physically and psychologically from attacks. Often, they were tasked with caring for others, when they themselves needed to recover physically, mentally, and emotionally. The psychological impact was a principal reason that some staff resigned from their posts and looked for work in areas that were more stable, taking time to recover from their experiences. The staff that remained in their posts were overburdened and under-resourced, which led to professional burnout and potential clinical errors. When staff did stay, many had feelings that their work was unappreciated by their patients and at times, they were even threatened by them. One said,

Well, you help everyone in the village, the destitute, the poor, the armed groups, you help them. Then they come and threaten you. Would that make you feel good? That's what I feel as a consequence. I got sick after the attack, and then I went back to work.

Healthcare workers continued to be affected by the constant threat of repeat attacks. Their distress was further exacerbated by the fear and demoralization they witnessed in their colleagues. One said:

We work in difficult conditions on the psychological side because we know that at any moment we can be attacked and … that influences the work climate and the attitude… if staff feel threatened and there are rumors that, in so many days they're going to be attacked; you see, no? Morally even, it's normal to be afraid and that’s part of [the difficulties] … So it necessarily affects the patients and the stress and the working conditions.

In addition, healthcare workers reported having less access to medications and supplies than before, working in damaged buildings, and even lacking bedding and other supplies. They lamented their inability to provide the quality of care they were trained to provide. In many cases, they could clinically diagnose conditions, but lacked the tools to confirm it. When they could properly diagnose, they often lacked the medications to treat them. Many expressed feelings of powerlessness to improve the situation or provide appropriate care for the population. Those in supervisory or supply positions could not do their jobs because of the impediments to travel.

As you know, immediately after this event, everyone withdrew to the [displacement] sites, and shortly afterwards, when people started to return home, there were many cases of illness, and we had no medication. When patients arrived at the hospital, they had nothing to sit on, and there weren’t even beds for [the very sick] to lie on...

While mentioned less frequently, some healthcare workers remained trapped at the facility or in a village or town which they were not originally from, because of the risk of being attacked during travel. They were unable to take their leave to return to their homes and spend time with family and friends. As a result, they often felt isolated, homesick, and demoralized in not knowing when they could see loved ones again. Their inability to take leave also led to burnout and its associated symptoms such as depression, loss of motivation, feelings of helplessness, isolation, self-doubt, and mood swings. One explained:

The damage was considerable, and it lasted. Even one of our staff wanted to go to the provinces, but he was harmed, and the [colleague] also suffered damage. He was on his way to [redacted] accompanied by a colleague from Bangui. On the way, they were intercepted by armed groups who robbed them of all their motorcycles and even their service telephones.

In many cases, staff and supervisors commented on the difficulty of balancing the necessity of providing health care to the population against the risk posed to staff. Personnel expressed the willingness to provide support, even within such a difficult context, but recognized that if they were also harmed, they would be unable to care for patients anyway. One said:

The consequences… are that we can no longer work there because it's already a question of safety. There was a shortage of staff…but if they're attacked, we can't continue to take the risk of bringing them into danger.

### Prevention and mitigation

#### Measures to increase security

National armed forces and the UN Multidimensional Integrated Stabilization Mission in the Central African Republic (MINUSCA) were occasionally helpful in securing areas after an attack, but primarily provided security only within their bases. In one case a MINUSCA base became the de facto health center until the situation stabilized and the health center was able to reopen. In some cases, staff moved large and valuable items like refrigerators and vehicles to MINUSCA bases. When they did secure an area after an attack, they reassured residents to go back to their homes and gave health center staff the confidence to reopen services. MINUSCA protection is limited, non-systematic and often took place hours or days after an attack had ended. One participant said:

[The hospital wasn’t closed] too long, about two weeks. The United Nations came, and we reopened the hospital.

Security, however, remained a persistent issue and staff frequently despaired of the lack of support they received. One respondent said:

Frankly, there's nobody here. No one could come. The problem was if the State was absent, we couldn't make any movements. I was able to send the news to the district and they said nothing. Even Bangui was informed. Absolutely no one could come. [We are] thankful for the arrival of IMC. They were informed, but they couldn't do anything. If it was the State that asked us, as you are doing, we would explain [the incident and its effects] to them, but it was all over like that…. They came to loot the village and the center and left, there was no one to talk to about it.

Prevention of attacks often required engagement with armed groups and a strong neutral stance regarding the parties engaged in conflict. Community members and local leaders were often effective in brokering accords with the groups. The willingness of healthcare workers to treat all patients also proved helpful. One participant told of a case where a community member negotiated ceasefire with members of armed group by saying that they should leave the hospital in peace so that they too could have the benefits of being cared for there.

They haven't touched the large regional hospital. They themselves are pleased that they too are treated in this hospital. At the beginning, they were only going to the hospital grounds to look for certain people in order to remove them and have them provide care elsewhere. But it was a pastor who stopped them, saying that a hospital is a place where you can't go in with a gun in your hand. And that's how they gave up. They don't act like they used to.

During vaccination campaigns, they often coerced staff to consult with them for safe passage or even to hire their members to help. While this practice compromised service independence, it also provided a level of safety for the staff. One participant said:

Vaccination campaigns are organized periodically, and generally when there is a vaccination campaign, we are obliged to approach the leaders of this armed group to make some of their [people] available to us, or we rent their motorcycles to give to the agents to do activities with. So it is mandatory that we involve them. If we involve them, they will secure the agents who go to the households or villages to vaccinate the children.

Cases where armed groups were directly involved in health activities were exceptional. More often, armed groups engaged in extortion and intimidation. They imposed “taxes” on health vehicles traveling on the road. Armed group presence in a facility, even for treatment, deterred people from seeking care and frightened patients. One participant said:

[In] February 2016 [the surgical ward] was not working well. All the patients were in anguish, since one [of the combatants] was injured. He spent 10 days in the hospital and his colleagues came in time to visit him. People were afraid.

Some mechanisms employed to protect communities also helped the health facilities. For example, some participants mentioned escape plans, addressing underlying religious tensions that fueled attacks, and community armed defense, especially with respect to groups from Sudan and Chad. A participant with extensive experience across the region described the desperation of communities to safeguard their health facilities. During attacks, they prioritized defending these facilities over other town areas, recognizing their lives often depended on the health care they provided. This was especially the case when the health facility was built by community members.

[The community suffered a lot because of lack of healthcare. Whenever they have a health facility, they really care about keeping it. They will defend whatever they have. Those health facilities are damaged only in extreme type of violence when the community can no longer defend it]

#### Mitigating the impacts on patients and the community

Community members and health care personnel alike showed enormous perseverance in the face of attacks. Participants cited community mobilization through the efforts of community health boards, community health workers, and health center staff, as the principal strategy to mitigate the impacts of attacks. These community members used their own personal resources and compromised their and their family’s safety, after suffering threats, violence, or the loss of loved ones or colleagues, to help the community recover. In some cases, health workers traveled to the bush to provide care to people who could not come to health centers.

I was the one who went out into the bush to treat them. Especially the little children, until everyone returned to the village.

One participant reported that community members donated their own money to buy a small supply of medication and materials while waiting for the facility to be restocked. Sometimes facilities limited treatment to conditions for which they had medications and appropriate technical training. Other facilities continued to provide consultations and where medications weren’t available, wrote prescriptions hoping patients could fill them elsewhere, mainly from sellers in the market or unofficial kiosks. To obtain them, though, patients had to pay out of pocket and often had to rely on unlicensed sellers and private pharmacies. One participant explained:

But most of the population preferred to be treated locally, as there were traders who sold a lot of medicines here locally, so prescriptions were given out and people bought medicines right here [in the streets].

Staff who came from the community where they worked were quicker to adjust after attacks; those who were from other parts of the country (credentialed personnel) were more inclined to leave. When credentialed personnel fled, local first aid and community health workers often stepped in to provide care. Remaining staff provided them with training. Some facilities had no interruption of services because of the dedication of local staff and community members. Others experienced only short-term gaps, organizing themselves so that services could be restarted as soon as it was possible. One said:

I recruited an agent to replace [the staff who left] and we recruited a woman and trained her to work in the place of the midwife. We trained a first aid worker, who took [the nurse’s] place, and then the assistant took the place of the head of the facility.

Participants spoke strongly about their determined advocacy efforts to secure aid, rehabilitate facilities, and restore services. Some were successful, receiving good support from local or district health system actors. Head nurses, supervisors, health officials and others responded to staff requests to travel to support their colleagues, advocate to replace staff that had fled, and make requests for stock from NGOs, the UN, and the MSP. They likewise applauded the dedication and bravery of their colleagues who remained in place and went to great lengths to continue providing care. One said:

Me, being a surgeon, I couldn't sit back, and watch women die in front of me, so I went out of my way to find what I needed to work. And I went down to town to ask the [health authority] to contact the medical NGOs to help us in this field because [this] is the only large center where [other] health posts refer serious diseases. This is why we cannot stand by and watch the difficulties pass before our eyes.

Participants noted informal adjustments to policies and practices. One change was a shift from providing patients with small amounts of medication, which assured regular monitoring and reduced possibility of stockouts, to increasing supplies provided to last longer. For people living with HIV, the MSP began to tell providers to give enough anti-retroviral medication to last 5–6 months to limit patient visits, reduce their travel and exposure to harm. Staff did the same for patients enrolled in outpatient therapeutic feeding programs for malnutrition, giving ready-to-use therapeutic food for a month or more, instead of the usual two weeks. The adjustment also ensured clients and patients could be treated if facility supplies were pillaged. For similar reasons, staff used a decentralization strategy, supplying health centers with materials they would normally only provide to referral facilities. Some health centers received emergency kits to conduct caesarian sections, when standard policy had required complicated deliveries to be referred.

During this period [after the attack], the number of referrals dropped significantly. Even malnutrition cases didn't come, and malaria cases didn't come either. [The staff] dealt with it at their level. After the incident, I heard Mr [redacted] operated on two pregnant women by Caesarean section, and there was no problem. He took the decision to do so because there was still a great risk [of attack]. Patients who weren’t referred, it's because of this attack.

Heads of health centers sought to reduce exposure to violence by minimizing the staff on shift, though this potentially compromised quality of care. One participant noted that while rural areas need qualified staff like doctors, their shortage means that losing them to an attack not only depletes the already fragile health system, but also reduces its capacity to train others. The MSP sometimes recalled credentialled personnel following attacks on healthcare. In efforts to preserve their safety, it also requested assurance from the community that the area was secure before sending highly trained staff.

Heads of health centers accompanied patients to referral facilities for services that were no longer available at their own facilities due to the effects of attacks, sometimes bringing them in their personal vehicles. Managers limited the inventory of supplies and medication at facilities and removed cash from them. This strategy reduced the risk of losing assets in the event of an attack though increased the risk of stockouts, particularly when movement was difficult due to insecurity or poor road conditions. Sometimes, if they knew an armed group would be passing through, they would send community members to hide supplies in a more secure location. Larger hospitals in the area sometimes held supplies for smaller facilities, restocking them with smaller amounts more frequently, or provided services that could no longer be furnished locally. One participant reported:

Children receive their vaccinations because the hospital sends staff to the health center for 2-3 days to vaccinate them and then bring the vaccines back.

International NGOs and UN agencies including International Medical Corps, Médecins Sans Frontières, Save the Children, the World Health Organization (WHO) and the United Nations Development Programme (UNDP) supported facilities after an attack, often replacing stolen medicine and materials.

After what happened, we turned to one of our partners, IMC. We talked to them, and they provided the health center with medicines to help the population.

Local NGOs also supported health structures after an attack, particularly when the population had fled the area and were living in sites for displaced persons. In some cases, local and international NGOs restarted services or offered direct care, such as through mobile clinics, including in places where displaced people were living, such as the bush.

There were limitations regarding these efforts, as international and local NGOs faced the same challenges regarding security and movement as the community and health center staff. In some cases, after an attack, NGOs suspended assistance, or pulled out of the area altogether, which left the community feeling despair and hopelessness. Some health professionals who could no longer work safely in their facilities moved to other locations. One NGO staff member reported relying heavily on the community to negotiate with armed groups and assure staff security. Some participants named the limited scope of donors, who only approved short-term aid for limited services, as a challenge to operating in insecure areas.

Participants also had a mixed view of governmental mitigation and support measures. One respondent credited the Ministry of Planning for signing agreements with international and national NGOs to provide direct services in these sites. Another mentioned the reluctance of the MSP to send credentialled personnel back to insecure areas following an attack. Others, however, said they had little support from CAR’s government, including the MSP and its regional and district health authorities, regarding resupply, facility repairs, and replacement of staff who had fled. They stated they had received little information from the MSP or agencies about help in restoring services, nor did someone come or call to ask about details of the attack.

No agents came, no NGOs, no government personnel to ask questions about the events. We, the children of the village, got together to get to grips with the situation and see what could be done, but there was no government presence, not even any projects, and there was no follow-up after the attack.

In part, these problems were a product of difficulties in communication and reporting. Many participants were unclear of the extent to which the government or UN were informed that an attack had happened at all or of the details. Poor phone and Internet connections and difficulty and insecurity of travel made reporting difficult. Sometimes travelers were asked to share the information at the central level but in those cases many details of the incident were likely to have been lost.

Reporting was also limited due to fear of reprisals from armed groups and participants said it was often not worth the risk of reprisals from reporting, especially if they had no confidence that reporting could improve security. Senior administrators in the provinces spoke of the difficulties they had in obtaining information about attacks. When attacks were reported, many spoke of informing supervisors in the MSP. Less commonly, attacks were reported to MINUSCA.

## Discussion

Our research in CAR reveals a complex and alarming landscape of attacks against health facilities, their staff, and patients. It underscores the deep and enduring impacts of such violence on both healthcare delivery and community well-being. The pervasive nature of attacks not only inflicted direct harm, but also led to serious operational disruptions, with protracted or permanent closure of services, dire shortages of staff and insufficient medical supplies. The aftermath of the attacks shows a health system struggling to meet the needs of the population amidst ongoing conflict.

Attackers looted medical supplies, cash, and medication, damaged, burned down or otherwise destroyed facilities, and threatened, killed, abducted, and physically or sexually assaulted staff. Some attacks appeared to be part of a wave of violence inflicted on a community, while others were specifically targeted at facilities and staff. The perceived reasons for targeted attacks varied: financial gain, personal use of goods, retribution for alleged poor quality of care or refusal to prioritize treatment of combatants, retaliation for treating enemy combatants, or as one element of a broader attack on the local population.

The acts of violence against health care in the three prefectures of the study (Haute-Kotto, Ouaka, and Vakaga) align with findings from studies in other countries at war, though there are notable differences compared to middle-income countries [20]. With some exceptions, looting is more common in chronic internal conflicts in low-income countries in Africa, notably in Burkina Faso, the Democratic Republic of the Congo, Mali, Nigeria, South Sudan, and Sudan [20] as well as the Central African Republic.

In middle-income countries, health systems were reasonably well-functioning and well-staffed before the war. The systematic impacts of attacks in those countries varied. More than 600 attacks on hospitals over a decade severely undermined the health system in northwest Syria [21]. In Ukraine, however, despite more than 1,000 Russian attacks on health care and the departure of many staff, the health system maintained relatively high functionality [10]. In the Central African Republic, as is the case in other low-income countries, pre-conflict conditions including extreme poverty, a weak economy, insufficient health workforce, poor infrastructure, and a legacy of colonialism and past conflict magnified the impact of violence against health care and impeded the ability to recover from it [15, 22–25].

### Impact of attacks

As a result of the looting, physical damage, and severe staff shortages from attacks on healthcare, facilities frequently faced prolonged delays in re-opening, sometimes spanning months or years; some never managed to re-open. For those that continued to function, replacement of stock was difficult due to the dangers of travel and risk of future attacks. Consistent with other study reports focused on CAR [26], this study observed that health facilities or their grounds became a place of refuge for people fleeing violence. This significantly strained facilities' operational capacity and safety, and increased hygiene and sanitation problems for the displaced persons. Rebuilding infrastructure takes time and investment that the prefectures lacked. In 2019, 18.6%, 36.0%, and 5.0% of the facilities were partially destroyed in Ouaka, Haute-Kotto, and Vakaga respectively [27]. An additional 18.6% and 8.0% of facilities were completely destroyed in Haute-Kotto and Vakaga. Our study indicated that challenges were greatest for health posts and health centers in more remote parts of the prefectures.

The absence or insufficiency of healthcare services severely jeopardizes population health, exposing individuals to heightened risks of sickness and death. Without reliable access to medical care, infectious diseases can escalate unchecked, conditions that are preventable or easily treatable develop into complicated cases, chronic conditions can worsen and become unmanageable, and women may die needlessly in childbirth. Ensuring robust healthcare infrastructure and services is critical to safeguarding public health and reducing the risk of avertible morbidity and mortality in a population.

The impacts of prolonged closures, staff shortages, and lack of medication and supplies on the population disproportionately affected the most vulnerable groups. The quality of care for services for children, the elderly, and patients needing regular medication, such as people living with tuberculosis and HIV/AIDS, suffered. Some facilities had no choice but to close reproductive health, malnutrition, HIV, and surgical services. Vaccinations were disrupted, likely contributing to infectious disease outbreaks [28]. Such barriers to care likely exacerbated existing inequalities and inequities, disproportionately affecting those living farther from facilities and those with limited economic resources.

In CAR, prior to the recent conflict, many facilities lacked staff with professional credentials. Moreover, it was extremely difficult to replace staff who fled after attacks, a problem amplified by the pre-existing challenges in hiring and retaining staff in remote health programs. Fear was a consistent struggle for healthcare workers and communities alike, as they did not know when attackers would strike again. In 2019, in the three prefectures of the study, there were just 35 nurses, 11 doctors, and 11 midwives [27]. CAR has 1.0 credentialled medical staff per 1,000 people, far below the African regional density of 1.5 staff per 1,000 people [29] and the WHO recommended 2.5 staff necessary to assure adequate coverage of primary health care [30]. Staffing of midwives may have been particularly difficult due to the combined risk of sexual assault for women and large gender equity gap, rendering women’s status lower in society [31].

For health workers, the psychological trauma of exposure to attacks was severe and long-lasting. Similar to findings of other studies, the trauma was aggravated in many cases as a result of living through multiple attacks [12, 15, 16, 32]. Some staff were deeply affected by the psychological trauma of their colleagues. Furthermore, as in Syria, they suffered moral injury from being unable to deliver the standard of care for which they were trained and felt duty-bound to provide and felt powerless to change their situation [12, 33].

### Mitigation strategies

To mitigate the challenges, providers and the MSP employed mitigation strategies that involved painful compromises. The MSP allowed for extended home-administration of treatments like anti-retrovirals for HIV and therapeutic food for malnutrition. The strategy mitigated the potential for loss of stock when transportation or facilities were attacked, but heightened the risks of inconsistent adherence, increasing morbidity and mortality. For conditions like HIV and Tuberculosis, inconsistent adherence increased the possibility of antimicrobial and treatment resistance. Facilities that adapted by minimizing on-hand inventory and cash, storing them at more secure locations reduced risk of loss but increased potential for stockouts. Reducing the staff on shift at a facility protected their safety, but likely compromised quality of care and increased risk of burnout.

The MSP hesitated to send credentialed staff back to insecure areas after an attack out of concern the country would lose a member of the small pool of trained medical personnel. However, their absence not only hindered the ability to diagnose and treat patients but precluded the possibility that they could train others. International NGOs frequently resupplied facilities in the wake of the attacks and supplemented staffing in facilities. Some of them established mobile clinics. In collaboration with the World Bank, the MSP attempted to resupply medications and address staffing shortages. Its capacity to provide support was hindered by communication challenges, difficulty accessing affected facilities, resource shortages, and by a lack of knowledge of attacks. Challenges persisted across the conflict-affected areas. Throughout the crisis, IMC was the only partner that has never left Vakaga prefecture.

### Community response

Communities, in collaboration with the health workers who were part of them, provided the bulk of the support to facilities after attacks. Community members filled gaps in staffing after experiencing violence and threats themselves, at risk to their own lives and their families. Locally recruited staff were reported to be likely to remain after an attack and seek support for the facility, while non-local workers often left. When health workers fled, facilities operated with community health workers, traditional birth attendants, or individuals with first aid training. Some were trained by credentialed staff, but quality of care inevitably suffered. Healthcare workers who remained after attacks extended services to locations where displaced people had sought refuge.

In some cases, the community increased security for facilities by arming themselves. Other times, they negotiated with armed actors. Engaging directly with armed groups is increasingly recognized as a key strategy for improving the security of health programs. However, this engagement requires caution, due to the volatility and risk of interaction with armed groups. This commitment should be treated with nuance given the volatile nature of the promises made. Some international organizations, among them the International Committee of the Red Cross, Central African Red Cross, and MSF, work with communities to foster engagement with armed actors. The willingness of NGOs to engage with armed groups often depends on context, skills, and relationships with national governments [34]. Many communities in the three prefectures engaged with armed groups, despite the risks, and without the option of evacuation that NGOs have. Our findings were consistent with a study by Barbelet et al., that found communities in CAR are often successful in engaging with armed groups, particularly when the groups are decentralized and have existing relationships within the community [35].

### MSP and international response

The MSP has instituted a 2022–2026 five-year plan for strengthening the health system, supported by the World Bank [36]. It is in part designed to mitigate the severe health staff shortages in conflict-affected regions of CAR. The plan includes salary supplements for personnel working in under-served areas and training to advance health worker skills. Some international NGOs and donors back these initiatives, in one case going as far as to establish a training school for health personnel outside of the capital. Results from project monitoring have indicated the strategy is promising for the future of the health system [37]. Although initiated after the study period, the strategy represents an important commitment from the MSP to support health facilities and personnel at risk of or affected by attacks and has engaged directly with armed groups. [38].

International donors have been hesitant to invest in the health system in recent years and their commitments to CAR generally waned further after the failure to negotiate a peace agreement in 2019 and the implication of the Wagner Group [39–41]. Poor governance and the lack of impact from previous funding may have also led some donors to reduce or pause support [37, 42]. In the two years following, the amount of aid to CAR dropped by $24 million [43]. In 2023 the amount of funding from donors for health was only 60% of the amount requested by the Health Cluster. Despite its extreme poverty, CAR now ranks 54th globally in receipt of development aid [44]. In 2023, moreover, cash and voucher assistance to individuals declined by 17% from 2022, attributed in part to the overall decline in assistance to CAR [45]. It should also be noted that of the funding granted to CAR, a small fraction has been used to the direct benefit of the population and even less has considered sustainability [37, 42].

MINUSCA, which has a strong mandate to protect civilians, played a modest rule in providing security to health facilities. In some situations, MINUSCA has stood idly by in the face of attacks by armed men, failing to protect the population in the face of danger. Participants reported that MINUSCA permitted staff to store equipment and supplies at their bases, and in one instance to deliver services there. However, aside from a few exceptions, like manning a checkpoint at a hospital entrance, it did not provide direct security to facilities. MINUSCA’s role in security can also be complicated by the religious or ethnic composition of the force [38]. This has been considered a factor which fosters partiality in response to a crisis with existing religious and ethnic tensions.

### Reporting and prosecution of attacks

Reporting and analysis of attacks, essential to improving security, remain deficient. Data collection is sporadic, reports are not disseminated to stakeholders, and information gathered is inadequate to develop strategies to enhance health security and mitigate the harmful impacts of attacks. The WHO Surveillance System for Attacks on Health Care (SSA) suffers both structural and operational defects that prevent it from fulfilling its mandate of documenting attacks [46]. The majority of MSP personnel do not even know of the existence of the SSA mechanism. Its reliance on data from the Health Cluster, which is not fully representative, limits its scope. It fails to share information with the national authorities, communities or local NGOs and does not provide decision-makers with details such as the location, timing, and nature of incidents [47]. Its design has not been adapted to the unique challenges of reporting in the Central African Republic and other countries experiencing protracted conflict. Further, as data are not used for protection or support, health providers have few incentives to report. During the 2016–2020 study period, WHO registered 82 attacks, 5 deaths, and 13 injuries across the 20 prefectures of CAR, compared to the 126 attacks, we identified in the three prefectures within our study area [46].

There has been impunity for attacks on health care worldwide, including an absence of prosecutions for war crimes committed against health facilities, staff and patients [38]. The International Criminal Court having jurisdiction over war crimes and crimes against humanity in CAR since 2012, declared in 2022 that it would not initiate further investigations beyond current cases, none of which involved attacks on health care [48]. Instead, the prosecutor emphasized cooperation with the Special Criminal Court established in 2015 to investigate and prosecute grave violations of human rights and the Geneva Conventions in CAR since 2023. This court, however, suffers from administrative slowness, difficulties linked to the constitution of evidence. These elements contribute to the disappearance of evidence and make prosecution difficult due to lack of evidence [49].

The Court, in cooperation with the UN, includes international and domestic judges, prosecutors, and administrators. Despite facing operational challenges to staffing, arrests of perpetrators, witness protection, funding, and security, the court has made progress, issuing its first verdict, for a massacre of 46 civilians in November 2022 [50]. In September 2023, it charged the former leader of a major armed group, the *Front Populaire pour la Renaissance de la République Centrafricaine,* for war crimes and crimes against humanity [51]. The charges did not address attacks on healthcare and many other actors, who have directly and indirectly contributed to human rights abuses remain at liberty.

### A way forward

Effective strategies exist to safeguard healthcare for the people of CAR. To protect healthcare from violence and assist the communities and health workers confronting it, a number of critical steps are needed. CAR’s government and international partners should increase support, assessing needs, increasing security, facilitating communication, and mitigating impacts of attacks by quickly rebuilding, resupplying, and re-staffing facilities. The international community must stop shirking its responsibilities to provide the financial aid and other support needed for health care in CAR. A sustainable approach, which bridges humanitarian efforts and development is the best way to build resilience within the health system. This includes resources for continuous training, psychosocial support for health workers and communities who have been harmed and are still on the front lines. As Barbelet et al. concluded, donors should invest in community organizations and civil society groups to strengthen community capacity to prevent violence and enhance resilience against it [35, 52].

Moreover, given the stated commitments of the MSP, donors beyond the World Bank should prioritize support for the 5-year plan, especially by funding health worker training and salaries. It is crucial to finance replacement of staff when they are forced to flee violence, and provide protection for front-line staff, recognizing the health system does not exist without them. Donors should further ensure that staff receive adequate compensation, training, and appropriate psychosocial support. Inconsistent support will only exacerbate suffering, perpetuate impunity, and neglect opportunities to alleviate consequences of the violence. Although poor governance has often been cited as the cause of the healthcare system's poor performance, very few, if any, of the programs proposed by stakeholders focus on setting up an effective, efficient and accountable system with a view to curb the ills that are devastating the Central African healthcare system.

Data collection should be explicitly linked to protection, reduction, and especially mitigation of risk. WHO should overhaul the SSA to ensure its effectiveness in CAR, incorporating new sources of data, improving data sharing with communities and stakeholders, enhancing reporting transparency, and collaborating effectively with the MSP, NGOs and communities to develop strategies to lessen the impacts of attacks.

Given the profound effects of violence against health care on both its availability to populations and on individual victims, the Special Court should prioritize the investigation and prosecution of crimes involving violence inflicted on health care. The International Criminal Court should further offer vigorous support to the Special Criminal Court in CAR.

### Limitations

This study focused on catchment areas within three prefectures of CAR, largely supported by international NGOs, and may not represent conditions in unsupported areas. The study likely obtained information about the most severe attacks. Given the frequency of attacks, delay between attacks, retention capacity, and data collection, respondents’ accounts are strongly subject to recall bias. The traumatic nature and psychological effects of the violence may likewise have had an impact on participants' recollection of events. Despite efforts to triangulate data with other sources, some details of attacks could not be corroborated. Many respondents were reluctant to share information because of fear of reprisals. Because threats were so common, they were often not mentioned and thus the study was unable to distinguish the effects of specific threats.

The study relied on key informant interviews with participants in public roles or employed by NGOs, excluding community members outside formal health or administrative positions. As a result, the study primarily examines the effects on the health system and service delivery, with impacts on patients and communities addressed indirectly. For the same reason, we did not capture information about violence and obstructions, such as roadblocks, that prevented individuals from obtaining care at facilities, unless they were known by key informants.

Carrying out interviews by telephone and Skype / Zoom presented its own challenges. Some interviews were affected by poor telephone or internet connectivity, with interviewers and interviewees having to repeat themselves, or resulting in dropped calls. This likely resulted in frustration on the part of the study participants and may have resulted in participants not going in as much depth during discussion of attacks, or not reporting some attacks they recalled, as they simply wanted to conclude the interviews.

Given the sensitive nature of interviews, positionality of interviewers could have affected the findings. Interviews were primarily carried out by researcher at ICASEES. The remote position of these researchers, all of whom were in Bangui, from the key informants, located in the three prefectures, is likely to have reduced potential concerns of key informants about the researchers’ position in the highly localized conflict in peripheral areas. Nevertheless, the role of ICASEES in CAR government may have discouraged some participants from sharing some information about attacks if they had distrust towards the government. Two non-Central African interviewers, affiliated with US and European academic institutions, also carried out some of the interviews. It is possible that some individuals interviewed felt more comfortable sharing information about attacks, in particular identity of attackers, in these interviews, if they considered these interviewers less likely to take an active view of the conflict or to align with particular armed groups. On the other hand, some interviewees may have felt reticent to share sensitive information with foreign individuals. Most of the interviews carried out by the two foreign interviewers were with foreign NGO personnel, which we believe would have mitigated some of the risk of this bias. We further cannot guarantee that the interviewers’ personal beliefs, ethnicity, and view of the conflict in the three prefectures did not influence the way that they carried out the interview. However, we did not identify any such obvious instances during analysis of interview transcripts.

Due to the gender inequity present in CAR [31], key informants were predominately male. Identifying key informants was challenging, and participation from female informants was further compounded by the underrepresentation of women in key positions within the CAR health system. This represents a significant limitation, as the perspectives and impacts on female health workers, are likely different from their male counterparts and findings here are described largely through a male lens. The study used an inductive approach to coding and analysis, which carries the risk of researcher bias as subjective interpretation can influence theme identification and findings. Nevertheless, the breadth and consistency of the accounts assure dependability to study’s findings.

## Conclusion

Violence inflicted on health care is carried out with impunity and is pervasive in regions of CAR affected by conflict. The psychological and physical toll of the attacks on providers and communities is profound, while the chronic instability and subsequent operational challenges have further destabilized a health system that has long suffered from insufficient quality and quantity of human and material resources. Widespread poverty and displacement, poor communication and infrastructure, political instability, unequal distribution of resources at decentralized level, and the limited capacity of the central government to prevent attacks or mitigate their impacts exacerbate these harms. Despite the difficulties, initiatives to support communities that bear the brunt of the violence, to enhance the capabilities and training of health workers, and to develop new strategies for protection, can reduce the suffering. A concerted effort from local and international stakeholders is essential to strengthen the health system in CAR, protect those on the front lines of care, and ensure the population has access to life-saving services.

### Supplementary Information

Supplementary Material 1

Supplementary Material 2

## Data Availability

The datasets generated and/or analyzed during the current study are not publicly available due to the sensitive nature of the content but are available from the corresponding author on reasonable request.

## Abbreviations

CAR: Central African Republic
ICASEES: Central African Institute for Statistics and Economic and Social Studies
IDP: Internally Displaced Person(s)
IMC: International Medical Corps
KII: Key Informant Interviews
MINUSCA: UN Mission in the Central African Republic
MSP: Ministry of Health and Population
NGO: Non-Governmental Organization
SSA: WHO Surveillance System for Attacks on Health Care

## Footnotes

1 The definition of in-hospital mortality takes into account all deceased people who have been brought to or died in the health facility and are confirmed dead by a healthcare worker.
