# Supplementary material for: “It's normal to be afraid”: attacks on healthcare in Ouaka, Haute-Kotto, and Vakaga prefectures of the Central African Republic, 2016–2020
Source: Confl Health. 2024 Aug 27;18:54. doi: 10.1186/s13031-024-00610-8 (PMC11351750; doi:10.1186/s13031-024-00610-8)
Supplement: Supplementary file 1 — Supplementary Material 1 [file 13031_2024_610_MOESM1_ESM.docx]

# Supplementary materials to “It's normal to be afraid”: attacks on healthcare in Ouaka, Haute-Kotto, and Vakaga prefectures of the Central African Republic, 2016-2020

Natalya Kostandova^*^, Jennifer Okeeffe^*^, Blaise Bienvenu Ali, Pierre Somsé, Audrey Mahieu, Odilon Guesset Bingou IV, Sebastien Dackpa, Gerard Mbonimpa, Leonard Rubenstein^+^

**Key informant interview guide (French)**

| **GUIDE D’ENTRETIEN POUR LES INFORMATEURS-CLES** |
| --- |

| FORMAT |
| --- |

- *Présentez-vous:* Bonjour, je suis (nom) et je suis chercheur à l’ICASEES, l’Institut Centrafricain des Statistiques et des Sciences Economiques et Sociales.
- Nous menons une étude en partenariat avec l’Université Johns Hopkins et l’Université de Genève. L’objectif de cette étude est de documenter les actes de violences contre les soins de santé, et de comprendre leurs effets sur le système de santé et sur la santé des populations. Je souhaiterais m’entretenir avec vous pour mieux comprendre les actes de menace, de violence physique ou verbale qui ont affecté les services de santé dans (votre région médicale / district sanitaire / la zone couverte par votre organisation) entre 2016 et 2020. Nous appelons attaques contre les soins de santé les attaques perpétrées contre les structures de santé, les agents de santé, les patients et les véhicules à usage médical.
- Je souhaiterais souligner le fait que l’entretien ne portera pas sur votre expérience personnelle ou la façon dont vous avez ressenti personnellement ces attaques. D’ailleurs, si vous avez été affecté directement par une attaque, je vous demanderai de me le signaler.
- *Si le formulaire de consentement a été envoyé par e-mail, demandez*: Avez-vous bien reçu et lu le formulaire d’information et de consentement? Avez-vous des questions?
- Je souhaite vous informer (1) que l’entretien durera environ de 45 min à 1 heure; (2) que le contenu de l’entretien restera confidentiel, c’est-à-dire que les informations que vous me révélez ne seront partagées avec personne d’autre si ce ne sont les membres de l’équipe de recherche; (3) que vous ne pourrez pas être identifié lorsque nous rapporterons les résultats; et (4) que vous pourrez vous retirez à tout moment de l’étude sans justification.
- Un enregistreur sera utilisé afin que toutes les informations partagées lors de l’entretien soient correctement capturées et retranscrites. L’entretien sera seulement enregistré avec votre accord.
- Avez-vous des questions?
- Consentez-vous à être interviewé?
- Autorisez-vous l’enregistrement de l’entretien?

| PROFILE DE L’INFORMATEUR CLEF |
| --- |

1. Titre et position **actuelle** de l’informateur-clé:
2. Sexe:
3. Titre et position qu’il / elle a occupé entre 2016 et 2020 dans la Vakaga, Haute-Kotto ou Ouaka:
4. De quand à quand a-t-il /elle occupé cette position?
5. Organisation pour laquelle il / elle travaillait?:

| GUIDE DES QUESTIONS D’ENTRETIEN |
| --- |

## Information sur les types d’attaques

1. Pourriez-vous lister les actes de menace, d’obstruction à l’accès aux soins, de violence physique ou verbale à l’encontre des agents de santé, des patients, des struture sanitaires ou des transports médicaux qui ont eu lieu depuis 2016 et dont vous avez connaissance(ou au cours de la période d’intérêt pour la zone en question)?

*Listez toutes les attaques dont le participant a connaissance et posez la question 2. S’il y a une attaque que vous souhaitez aborder en particulier, relancez “Nous avons entendu parler de [attaque]” et posez ensuite la question 2. Sinon, commencez par la première attaque listée par le participant.*

1. Vous souvenez-vous de la date à laquelle l’attaque a eu lieu?

Si vous ne vous souvenez pas de la date exacte, vous rappelez-vous la période à laquelle l’attaque a eu lieu?
Suggérez:

- Était-ce aux alentours d’un grand événement ou d’une période de congés?

*Référez vous au calendrier des évènements pour guider les participants s’ils ne se souviennent pas de la date exacte*

1. De quelle manière avez-vous été informé de cette attaque?
2. Avez-vous vous même subi des violences au cours de cette attaque?

Si oui, demandez s’il connaît quelqu’un qui n’a pas été directement affecté par cette attaque et qui pourrait nous renseigner dessus, et passer à la prochaine attaque. Ne posez pas de question sur cette attaque.

1. Pouvez-vous décrire ce qui s’est passé?

Suggérez:

- Où l’attaque a-t-elle eu lieu? (Demandez le nom de l’air de santé, le nom de l’hôpital de district et du poste de santé, le nom des ONGs actives dans cette zone (si pertinent)).
- De quel type d’attaque s’agissait-il?
  - Intimidation, harcèlement, arrestation, détention, ou enlèvement d’agents de santé, forcer les personnels de santé d’agir conre leur éthique; bombardement, explosions, pillages, vols, détournement de véhicules, fussillade, tirs de feu, fermeture forcée des structures sanitaires, incendie, incendie volontaire, usage de la force militaire à l’encontre des principes humanitaires, déni ou retard de prise en charge du patient, assaut, exécution, torture, violence sexuelle, violence psychologique, obstruction, menace d’usage de violence, autre (veuillez décrire en détail).
- Qui était impliqué dans cette attaque?
  - Les perpétrateurs (*étaient-ils membres de groupes armés? Le participant ne doit pas être poussé à donner le nom du groupe armé, à moins qu’il le dévoile sans qu’on l’invite à le faire*), combien étaient-ils
  - Envers qui était dirigée cette violence? Ici, à nouveau, nous ne cherchons pas à connaître le nom des individus, mais plutôt leur rôle / description générale, ex: infirmière d’une NGO, agent de santé communautaire, patients cherchant à rejoindre une structure sanitaire
  - Etaient-ils des accompagnants?
- Savez-vous si cette attaque a été rapportée?
  - Si oui:
    - A qui cette attaque a été rapportée?
    - De quelle manière cette attaque a-t-elle été rapportée?
    - Savez-vous s’il y a des témoins de cette attaque?

## Information sur l’impact des attaques

1. Quelles furent les conséquences de ces attaques?
2. Y-a-t-il eu des blessés? Y-a-t-il eu des morts?

Suggérez:

- - Parmi les agents de santé?
  - Parmi les patients?
  - Parmi les personnes se trouvant dans la structure sanitaire?
  - Parmi d’autres personnes? (Par exemple, les personnes se rendant à la structure sanitaire; les membres de la communauté qui ont tenté de s’interférer, la population en général)

1. Quelle a été l’étendue des dégâts?
2. Est-ce que la structure sanitaire, l’équipement médical, le matériel, les fournitures, les médicaments, ou le transport médical ont été endommagés?

Suggérez:

- - Y-a-t-il eu du matériel médical ou des équipements volés ou détruits? Si tel est le cas, quel type de matériel? Comment cela a-t-il affecté le fonctionnement des services de santé?
  - Des médicaments ont-ils été volés? Quels types de médicaments? Comment cela a-t-il affecté le fonctionnement des services de santé?
  - Les moyens de transports (ex: ambulance) ont-ils été volés ou endommagés? Comment cela a-t-il affecté le fonctionnement des services de santé?
  - De l’argent a-t-il été volé? Comment cela a-t-il affecté le fonctionnement des services de santé?

1. Quel a été l’effet de cette attaque sur la disponibilité des services de santé?
   - Quels services ont-ils été interrompus? De quand à quand?

*(Note: Si le participant ne se souvient pas des dates exactes, essayez d’obtenir autant d’informations que possible sur les dates exactes en utilisant un calendrier des évènements, pour spécifier l’année, le mois et les semaines). Il est possible que les participants aient besoin de se référer à leurs registres. Dans ce cas, décidez avec eux d’une date de suivi pour obtenir l’information. Le même procédé s’applique au reste des questions “De quand à quand”, ci-dessous. Investiguez pour savoir si ce fût un changement graduel ou abrupt, avec le retour partiel des services après une certaine période, ou une interruption ou une reprise de services de manière abrupte.*

- - La structure sanitaire a-t-elle fermée? Si oui, combien de temps? (De quand à quand?)
  - La qualité des services a-t-elle été affectée? Si oui, de quelle manière? Pourquoi? Combien de temps? De quand à quand?
  - Ces attaques ont-elles eu pour conséquences des manques au niveau des ressources humaines? (ex: des agents de santé qui quittent ou se relocalisent suite à l’attaque). Combien de temps ces manques ont-ils persisté? De quand à quand? Si les agents de santé originels n’ont pas repris leur service, ont-ils été remplacés par de nouveaux agents de santé? Ont-ils été remplacés par une personne qui a une formation et des qualifications similaires (ex: sage-femme vs. accoucheuse traditionnelle; infirmier vs. secouriste)? Est-ce que les remplaçants prodiguent les mêmes services de santé que ceux qui ont quitté?
  - Est-ce que les agents de santé de la structure sanitaire / de l’ONG affectée par l’attaque parlent toujours de l’attaque?
    1. Comment l’attaque a-t-elle affecté le recrutement, le déploiement et les effectifs sur le long-terme?

*Suggérez: au niveau des structures de santé publiques, demandez les effets sur l’affectation du personnel du MSP et de l’ONG*

- - 1. Comment la santé mentale des agents de santé a-t-elle été affectée?

1. Quel a été l’effet de cette attaque sur l’accès et l’utilisation des services de santé?

- Si la structure de santé est restée ouverte, y a-t-il eu des changements dans la façon dont la population accèdent ou utilisent les services de santé (ex: les patients refusent de passer la nuit à la structure sanitaire)?
- Y-a-t-il eu des groupes plus affectés que d’autres? (ex: les groupes ethniques, les femmes, les adolescents, les personnes vivant avec un handicap)
- La population a-t-elle cessé de fréquenter la structure sanitaire?
- La population a-t-elle cessé de fréquenter certains services de santé? - Lesquels? Les gens ont-ils écourté leurs séjours dans la structure sanitaire? Ont-ils repoussé à plus tard leur visite à la structure sanitaire (ex: en venant seulement s’ils avaient des complications sévères)?

Pour chacun des services ci-dessous notez de quand à quand ces changement ont été observés.

- Quels services ont été affectés par un changement dans l’accès ou l’utilisation des services de santé?
- Avez-vous noté un changement dans le nombre de femmes fréquentant la consultation prénatale? De quand à quand?
- Avez-vous noté un changement dans le nombre de femmes accouchant à la maternité? De quand à quand?
- Avez-vous noté un changement dans le nombre de césariennes? De quand à quand?
- Avez-vous noté un changement dans le nombre de consultations pédiatriques (moins de 5 ans)? De quand à quand?
- Avez-vous noté un changement dans le nombre d’enfants fréquentant les services de pesée? De quand à quand?
- Avez-vous observé un changement dans le nombre d’enfants vaccinés? De quand à quand?
- Avez-vous observé un changement dans le nombre de personnes vivant avec le VIH qui reçoivent un traitement antirétroviral (si le service était toujours disponible)? De quand à quand?
- Avez-vous observé un changement dans le nombre d’enfants atteints de paludisme? Qu’en est-il pour les cas de paludisme sévère plus précisément? De quand à quand?
- Avez-vous observé un changement dans le nombre d’enfants traités pour malnutrition aiguë sévère? De quand à quand?
- Avez-vous observé un changement dans le taux de mortalité intra-hospitalière? De quand à quand?
- Avez-vous observé ou avez-vous eu écho de changement dans la morbidité ou mortalité au niveau de la communauté qui pourraient être une conséquence des attaques? De quand à quand? Pourriez-vous décrire ce changement?
- La population discute-t-elle toujours des attaques?

1. La population a-t-elle eu accès à une autre structure sanitaire pendant l’interruption des services de santé?
   - Quelle(s) autre(s) structure(s) sanitaire(s)?
   - Où cette autre structure sanitaire est-elle située? A quelle distance est-elle située de la structure sanitaire?
   - Cette autre structure sanitaire a-t-elle été inaccessible par moment?
   - La population a-t-elle utilisé les services de santé offerts par cette autre structure sanitaire?

Suggérez:

- Y-a-t-il des sous-groupes de la population qui n’ont pas eut accès à cette autre structure sanitaire?

**Si oui**, quels groupes de population? Pour quelles raisons? Ont-ils eu accès à des soins offerts ailleurs? (y compris des structures non-étatiques)? Où? Comment pensez-vous que cela a affecté ceux qui nécessitaient des soins et leur état de santé?

1. Des stratégies ont-elles été adoptées pour mitiger les effets de cette attaque? Si oui, pouvez-vous décrire ces stratégies?

Suggérez: quelles stratégies ont mis en place les ONGs, le MSP, les agences de développement, la société civile?

1. Y-a-il quelqu’un d’autre que vous recommanderiez et que nous pourrions interviewer pour nous parler de cette attaque?

*Précisez l’attaque sur laquelle l’expert pourrait fournir des informations.*

1. Cette attaque était-elle une attaque isolée ou faisait-elle partie d’une série d’attaques répétées?

Suggérez: quelles étaient les attaques répétées?

*Retournez à la liste d’attaques identifiées dans la question 1. Répéter les questions de sections II.2-5 et III.*

1. Y-a-t-il d’autres attaques auxquelles vous pensez?

*Si oui, répéter les questions de sections II.2-5 et III.*

## Information sur les évènements périodiques

1. Entre 2016 et 2020, au moment où ces attaques ont eu lieu, y-auraient-ils d’autres évènements qui auraient pu influencer la disponibilité, l’accès et l’utilisation des services de santé? Listez les et décrivez les un par un.

*En fonction de la liste d'événements que nous aurons collecté avant cet entretien, nous pourrions suggérer une liste d'événements. Pour chaque évènement, il est crucial de savoir QUAND l'événement a eu lieu et combien de temps il a duré. Si possible, les dates devraient être vérifiées; si ce n’est pas le jour exact, du moins les semaines au cours desquelles il a eu lieu. En plus des dates, demandez ce qui s’est produit, quels services ont été affectés, si certains groupes de populations ont été affectés plus que d’autres.* ***Si oui****, quels groupes de population? Pour quelles raisons? Ont-ils eu accès à des soins offerts ailleurs? (y compris des structures non-étatiques)? Où? Comment pensez-vous que cela a affecté ceux qui nécessitaient des soins et leur état de santé?*

*Suggérez la liste suivante:*

- Les ruptures de stock de vaccins (demandez quand les ruptures de stock ont eu lieu et quels vaccins)? Si oui, quel service de santé cela a-t-il affecté? Combien de temps?
- Perturbation de la chaîne de froid? Si oui, quel service de santé cela a-t-il affecté? Combien de temps?
- Les campagnes de vaccination (demandez quand celles-ci ont eu lieu, et le type de campagne)
- Congés annuels ou quelconque congé des agents de santé (demandez quels agents de santé étaient en congé, quand, combien de temps, s’ils ont été remplacés pendant cette période)
- Evènements (grèves, élections) Si oui, quel service de santé cela a-t-il affecté? Combien de temps?
- Afflux massif de patients dûs au conflit armé (demandez quand cet afflux a eu lieu, si les patients ont afflué d’autres aires de santé ou bien de la même aire de santé)
- Perturbation dans l’accès par la route aux structures sanitaires (demandez quand, pourquoi, combien de temps les perturbations ont duré)
- Attaques au sein de la communauté (demandez quand, quel type d’attaques, que s’est-il passé après les les attaques / comment pensez-vous que cela ait pu affecter l’accès et l’utilisation des services de santé)
- Déplacement de populations (quand les populations ont-elles été déplacées, venant d’où, déplacées vers où, combien de personnes)
- Population fuyant le conflit *(y compris quelles populations, venant d’où, fuyant vers où, combien de personnes)*

1. *Lorsque ces évènements autres et les attaques contre les soins de santé ont eu lieu au cours de la même période demandez:* Lesquels ont-eu le plus d’influence sur l’accessibilité, disponibilité et uitilisation des services de santé? Comment estimeriez-vous l’influence des attaques sur la disponibilité et l’utilisation des services de santé?
2. *En cas d’attaques multiples, demandez:* Quelles attaques ont eu plus d’influence que les autres / ont été plus difficiles à mitiger

_______________________________________________________________

Notez toutes les sections qui nécessitent un suivi (ex: les dates que le participant doit vérifier pour les attaques ou les évènements, les nombres), y compris en quoi consiste le suivi et la date butoire pour obtenir les informations.

Remerciez le participant pour sa participation!
