# Supplementary material for: “It's normal to be afraid”: attacks on healthcare in Ouaka, Haute-Kotto, and Vakaga prefectures of the Central African Republic, 2016–2020
Source: Confl Health. 2024 Aug 27;18:54. doi: 10.1186/s13031-024-00610-8 (PMC11351750; doi:10.1186/s13031-024-00610-8)
Supplement: Supplementary file 2 — Supplementary Material 2 [file 13031_2024_610_MOESM2_ESM.docx]

« C’est normal d’avoir peur » : attaques contre les soins de santé dans les Préfectures de Ouaka, Haute-Kotto et Vakaga de la République Centrafricaine, 2016-2020

Natalya Kostandova^1*^, Jennifer OKeeffe^1*^, Blaise Bienvenu Ali^2^, Pierre Somsé^3^, Audrey Mahieu^4^, Odilon Guesset Bingou IV^2^, Sebastien Dackpa^2^, Gerard Mbonimpa^5^, Leonard Rubenstein^1+^

1 Johns Hopkins Bloomberg School of Public Health, Baltimore, MD, USA

2 Institut Centrafricain des Statistiques et des Etudes Economiques et Sociales, Bangui, République Centrafricaine

3 Ministère de la Santé et de la Population, Bangui, République Centrafricaine

4 Geneva Centre of Humanitarian Studies, University of Geneva, Geneva Switzerland

5 International Medical Corps, Bangui, Central African Republic

*Co-premiers auteurs

^+^Auteur correspondant

## Extrait

##### Introduction

##### Les attaques contre les soins de santé ont encore affaibli le système de santé déjà fragile en République Centrafricaine. Nous avons investigués les attaques contre les soins de santé dans trois préfectures touchées par le conflit - Ouaka, Haute-Kotto et Vakaga - de 2016 à 2020. L'objectif de l'étude était d'acquérir une compréhension approfondie des effets immédiats et à long terme des attaques sur le personnel de santé, les structures, la chaîne d'approvisionnement, la qualité des soins et d'autres composants du système de santé. Nous fournissons une description qualitative des incidents, évaluons leurs impacts, identifions les efforts d'atténuation et discutons des défis à relever pour le futur.

##### Méthodes

##### Nous avons fait recours à la méthode d’échantillonnage raisonné et en boule de neige pour sélectionner les informateurs clés de l'étude. Des entretiens semi-structurés avec des informateurs clés ont été menés auprès des autorités administratives et sanitaires, du personnel de première ligne des formations sanitaires et des Organisations Non Gouvernementales (ONG). Les entretiens ont été effectués en Sango, Français ou Anglais. Les entretiens enregistrés étaient transcrits et des notes ont été prises pour les entretiens non enregistrés. Les transcriptions et les notes ont été analysées à l'aide d'un codage inductif, permettant aux réponses des participants de guider les résultats.

##### Résultats

##### Sur les 126 attaques identifiées au cours de la période d'étude, 36 informateurs clés ont discuté de 39 attaques. Les attaques comprenaient des meurtres, des agressions physiques et sexuelles, des enlèvements, des incendies criminelles, des bombardements à la grenade, des pillages, des occupations et des menaces verbales. La violence a entraîné des fermetures prolongées et des pénuries importantes dans les services de santé, affectant de manière disproportionnée les populations vulnérables, comme les enfants moins de cinq ans, les personnes âgées, les personnes avec des maladies chroniques, et les déplacées. Les travailleurs de la santé ont subi des traumatismes psychologiques et des préjudices moraux en raison des attaques répétées et de l'incapacité à fournir des soins adéquats. Le personnel de santé et les communautés ont déployé d'énormes efforts pour atténuer les impacts et plaider en faveur d'une assistance. Ils ont été limités par des mécanismes de signalement défaillants, une insécurité persistante et un manque continu de ressources et de soutien externe.

##### Conclusion

Il existe des stratégies efficaces pour protéger les soins de santé de la violence, mais il est essentiel de mieux soutenir les communautés et les travailleurs de la santé, notamment par des mesures visant à évaluer les besoins, à renforcer la sécurité et à faciliter le rétablissement en reconstruisant rapidement les formations sanitaires (FOSA), en les réapprovisionnant en intrants et en les dotant de nouveaux personnels. Le Gouvernement Centrafricain, les organisations internationales et les bailleurs de fonds devraient déployer des efforts concertés pour améliorer les mécanismes de signalement et mettre fin à l'impunité des auteurs de violences. Leur investissement dans les organisations communautaires et le soutien à long terme du système de santé, notamment la formation du personnel de santé, les salaires et les soins psychosociaux, sont des étapes essentielles pour renforcer la résilience et atténuer les impacts des attaques contre les soins de santé.

### Mots clés

République centrafricaine, Conflits armés, Personnel de santé, Établissements de santé, Personnel de santé, Accessibilité des services de santé, Violence, Santé publique, Violations des droits de l'homme, Attaques contre les soins de santé

## Introduction

La République centrafricaine (RCA), l'un des pays les plus pauvres du monde, est en proie à un conflit interne depuis plus d’une décennie. Divers groupes armés non étatiques et constitués en majorité d’étranger contrôlent la majeure partie du pays et s'attaquent aux populations locales. Des évaluations ont estimé que dix des vingt préfectures du pays, regroupant 47,5 % de la population, étaient partiellement ou entièrement contrôlées par des groupes rebelles en juillet 2021(1). Le pays se classe au 191^ère^ rang sur 193 pays selon l'indice de développement humain des Nations Unies(2). La mortalité maternelle et infantile figure parmi les cinq pires au monde, le paludisme et la malnutrition sont très répandus(3). Plus d'un million de personnes sont déplacées et la moitié de sa population (de 6,1 millions d'habitants) a besoin d'une aide humanitaire(4). Les personnes vivant dans des sites des déplacés n'ont souvent pas accès aux services de base, notamment à la nourriture, à l'eau potable et à l'assainissement(3).

Le système de santé qui était déjà faible avant le conflit, s'est dégradé depuis par suite des dégâts subis pendant le conflit. Les dépenses de santé du gouvernement dépassaient à peine 3,50 dollars par personne et par an en 2023(5). Seule la moitié des formations sanitaires (FOSA) sont pleinement fonctionnels et il y a une grave pénurie de personnel qualifié, avec un ratio de 0,6 médecin pour 100 000 habitants, l'un des plus faibles au monde(6). Plus de 280 organisations locales, internationales et agences des Nations Unies opèrent dans le pays et apportent une appui à la population et au système national de santé, notamment en fournissant du personnel et en soutenant les structures publiques de santé(7). Si ces efforts sont essentiels pour sauver des vies à court terme, ils ne peuvent pas répondre aux besoins de santé de l'ensemble de la population ni se substituer à un système de santé durable, en particulier dans les régions du pays touchées par le conflit et, où les besoins en matière de santé sont les plus importants.

Le système sanitaire a été encore plus accablé par les attaques violentes et les menaces contre les formations sanitaires (FOSA) et le personnel pendant les conflits. Bien que les rapports sur les attaques soient limités et irréguliers, la Coalition pour la protection de la santé en temps de conflit (Safeguarding Health in Conflict Coalition), a fait état de 52 attaques contre les soins de santé en 2017 et de 47 en 2018(8). L'impact de la violence sur les soins de santé dans les conflits a fait l'objet d'une attention croissante au cours de la dernière décennie, mais à l'exception de l'impact sur la santé mentale, les études évaluées par des pairs ont principalement été menées dans des pays à revenu intermédiaire(9, 10) ; ou dans les pays à faible revenu et touchés par des conflits dans le Moyen-Orient(11-13). Dans les pays à faible revenu touchés par conflits dans les pays Subsahariens, où les services de santé étaient souvent faibles avant le conflit, à quelques exceptions près(14), les informations sur les attaques sont limitées aux données collectées pendant des brèves enquêtes, comme celles menées au Sud-Soudan(15) et au Nigéria(16), et des études qualitatives sur la violence au sein de la famille, de la communauté et des patients en République démocratique du Congo(17).

Trois préfectures touchées par conflit étaient considérées pour cette étude : Haute-Kotto, Ouaka et Vakaga. Elles étaient appuyées par le ONG International Medical Corps (IMC) et étaient retenu pour l’étude parce que IMC a pu faciliter le contact avec les participants, l’accès aux données associées et aider à la triangulation des résultats. De 2016 à 2020, les groupes armés ont été très présents et actifs dans ces trois préfectures (Figure 1). Les populations de Haute-Kotto, Ouaka et Vakaga ont été à plusieurs reprises exposées à de graves violences et à des déplacements forcés. Bien que de nombreux acteurs humanitaires aient été présents dans les préfectures, leurs activités se sont concentrées dans et entourant les principales villes de Bambari, Birao et Bria. De grandes parties des préfectures et de leurs villes majeures étaient engagées dans un conflit actif. Pour comprendre les effets des attaques contre les soins de santé sur les patients, les communautés et les prestataires dans les trois préfectures, nous avons mené une étude avec des méthodes mixtes en utilisant les entretiens clés pour l’étude qualitative et série chronologique interrompue pour la partie quantitative. Les objectifs de l'étude étaient d'évaluer les impacts immédiats et à long terme des attaques, d'identifier les efforts d'atténuation menés par les communautés et les partenaires, et de décrire les défis importants auxquels sont confrontés les communautés, les prestataires et les autres acteurs pour rétablir l'accès et la qualité des services de santé. Nous présentons ici le volet qualitatif de l'étude.


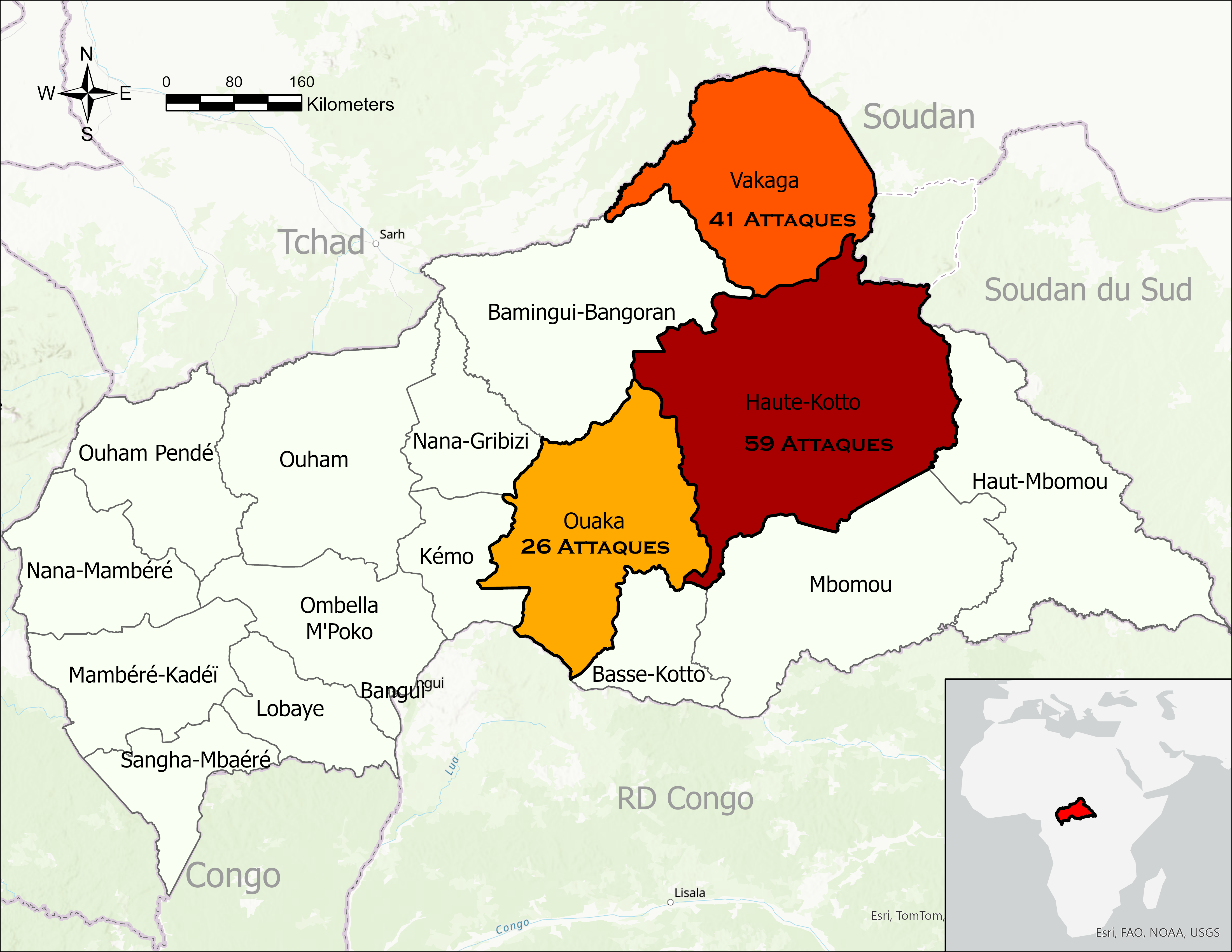


Figure 1. Attaques contre les soins de santé dans les préfectures de Haute-Kotto, Ouaka, and Vakaga, RCA, 2016-2020, identifiés à travers de collecte des données primaire et sources des données secondaires

## Méthodologie

### Conception de l'Étude

Nous avons pris en compte les impacts physiques, psychologiques et opérationnels des attaques dans l'étude. Nous avons utilisé des méthodes qualitatives en nous appuyant sur des entretiens avec des informateurs clés pour analyser l'impact des attaques sur les soins de santé. L'étude est une initiative de consortium *Researching the Impact of Attacks on Healthcare(18)* avec la collaboration de l’Université Johns Hopkins Bloomberg School of Public Health, de la Ministère de la Santé et de la Population (MSP), de l'Institut Centrafricain des Statistiques et des Études Économiques et Sociales (ICASEES) et du Centre d'Études Humanitaires de l'Université de Genève.

### Cadre et Période de référence de l’Étude

Toutes les attaques contre les soins de santé dans les préfectures touchées par le conflit Haute-Kotto, Ouaka et Vakaga en République Centrafricaine de janvier 2016 à décembre 2020 étaient considérées pour l’étude. Initialement, l’étude a limité l’inclusion aux participants connaissant des attaques dans les zones de responsabilité de l’IMC. Cependant, lors des entretiens, les participants ont identifié des attaques dans les trois préfectures en dehors des zones appuyés par IMC, qui ont été aussi incluses pour leur pertinence par rapport aux objectifs de l'étude. Bien qu'il y ait eu des attaques généralisées contre la population générale ou d'autres institutions dans les trois préfectures, ces informations ont été exclues de l'analyse car elles sortaient du cadre de l'étude visant à étudier l'impact des attaques sur les soins de santé en RCA.

### Échantillonnage et Participants

Nous avons fait appel à des informateurs clés de IMC et MSP pour dresser une première liste de participants à l'étude, composée d'autorités administratives, de autorités sanitaires de districts et régionale, de prestataires travaillant dans les FOSA de MSP soutenues par l'IMC, et de membres du personnel du programme IMC. Les participants initiaux ont été sélectionnés sur la base d'une connaissance préalable des attaques individuelles survenues dans la zone et au cours de la période étudiées. Nous avons ensuite utilisé un échantillonnage en boule de neige pour identifier d'autres participants susceptibles d'avoir des informations sur les attaques spécifiques en raison de leurs positions professionnelles. Une attaque contre les soins de santé a été définie pour les participants comme ‘des actes de menace, de coercion ou de violence physique ou verbale, qui entravent la fourniture de soins de santé. Ces attaques peuvent cibler le personnel de santé en service, les patients, les FOSA ou les transports sanitaires.’ Tous les participants qui avaient connaissance d’une attaque spécifique contre les soins de santé au cours de la période d’étude et qui ont donné leur consentement éclairé ont été inclus dans l’étude. Nous avons exclu les participants qui avaient connaissance d'attaques uniquement en dehors de la période d'étude ou lorsque les soins de santé n'étaient pas directement ciblés. Les participants étaient des autorités administratives de la RCA, des prestataires de santé de première ligne du MSP, des directeurs régionaux et de district de la santé et du personnel des ONGs.

### Collecte des données

### Les enquêteurs et les transcripteurs de l'ICASEES ont participé à une formation de recherche en présentiel de trois jours qui couvrait le processus de consentement éclairé, les premiers secours psychologiques, l'éthique de la recherche, les compétences des enquêteurs, le contenu du guide d'entretien et les entretiens pilotes. Les enquêteurs ont mené des entretiens semi-structurés avec tous les participants à l'aide d'un guide préétabli (*Supplementary materials)*. Nous avons expliqué l'objectif de l'étude à tous les participants potentiels et décrit leur droit de se retirer de l'étude à tout moment. Les enquêteurs ont cherché à obtenir un consentement écrit mais ont accepté un consentement verbal lorsque la distance ou les problèmes de connexion Internet ne permettaient pas d'obtenir un consentement écrit. En outre, nous avons noté qu'aucune information permettant d'identifier les participants n'apparaîtrait dans les transcriptions, les résumés ou les manuscrits, en limitant les identifiants personnels à la voix lors des entretiens.

### La majorité des entretiens ont été menés par l'ICASEES, le reste par d'autres membres de l'équipe de recherche (NK + AM). Les entretiens ont été menés dans la langue choisie par le participant, à savoir, le Sango, le Français ou l'Anglais. Tous les entretiens enregistrés ont été transcrits en français pour l’analyse. Pour les participants qui ont refusé l'enregistrement, des notes ont été prises en anglais ou en français pendant les entretiens. Les entretiens ont été réalisés en distance par téléphone ou à l'aide d'un logiciel d'appel crypté (Skype, Zoom). Les enregistrements des entretiens ont été supprimés une fois la transcription terminée. Les transcriptions et les notes ont été stockées dans des dossiers cloud cryptés et protégés par mot de passe, accessibles uniquement à l'équipe d'étude.

La collecte de données s'est déroulée en trois phases : mai-juin 2022, décembre 2022-janvier 2023 et juin-août 2023. Les retards initiaux étaient dus à des ajustements opérationnels et à une formation supplémentaire. Dans la phase finale, l'équipe a assuré une couverture complète en interrogeant des participants qu'elle n'avait pas pu atteindre pour avoir la saturation.

### Analyse de Données

Tous les entretiens transcrits et les notes ont été analysés en anglais à l'aide du logiciel NVivo version 14.23.3 (61)(19) en utilisant une approche de codage inductif(19). Le codage inductif a permis aux réponses des participants de guider le développement des thèmes, fournissant une catégorisation des résultats basée sur les données. Un premier codage ligne par ligne, sans restriction, a été effectué sur un sous-ensemble de quatre transcriptions afin de développer des codes préliminaires (JO). Ceux-ci ont ensuite été édités, révisés, complétés ou supprimés pour refléter les données des transcriptions suivantes. Ce processus itératif a permis de s'assurer que l'ensemble final de thèmes été représenté fidèlement. Pour accroître la rigueur dans la fiabilité et la confirmation des résultats, un sous-ensemble de quatre transcriptions (>10 %) a été recodé par un deuxième membre de l'équipe de recherche (NK). Les différences ont été discutées entre l’équipe.

L'importance des thèmes a été déterminée à la fois par la fréquence des mentions dans les différents entretiens et par la profondeur de la description faite par le participant. La fréquence des mentions dans les différentes transcriptions n'a pas été prise en compte pour déterminer l'importance des thèmes. La double importance accordée à la fréquence des mentions et à la profondeur des transcriptions a permis d'identifier les thèmes les plus importants pour les participants et de s'assurer que les résultats étaient fondés sur leurs expériences.

Pour compléter l'analyse thématique, nous avons créé une matrice de données complète. Cette matrice a permis d'extraire et d'organiser les informations clés de chaque transcription, en fournissant une vue d'ensemble structurée des données. La matrice comprenait des variables telles que le lieu et la date de l'attaque, la position du participant au sein du système de santé, une description détaillée de l'attaque, son impact immédiat et ses effets à moyen et long terme. En outre, la matrice a pris en compte les effets des attaques sur les FOSA voisines, notamment l'absorption des patients des établissements attaqués, ainsi que les facteurs externes susceptibles d'avoir exacerbé l'impact des attaques, tels que les difficultés de la chaîne d'approvisionnement, qui ont entravé la capacité de réapprovisionnement de l'établissement après son pillage.

### Approbation Ethique

L'étude a été approuvée par le Comité éthique et scientifique de l'Université de Bangui, République Centrafricaine (N°27/UB/FACSS/IPC/CES/021), par la Commission d'éthique Universitaire de l'Université de Genève (CUREG.202101.11), et par le comité éthique de l'Université Johns Hopkins (Avis de détermination FWA #00000287). L'étude a reçu le Quitus statistique du Comité Technique de Programmation des Activité Statistiques (CTPAS), République Centrafricaine (N°0377/2022/MEPC/DIRCAB/ICASEES/DMNER).

## Résultats

### Entretiens

Au total, 126 attaques sur la zone et la période étudiées ont été identifiées. Parmi ces attaques, 41 entretiens ont été menés, dont 36 répondaient aux critères d'inclusion. Cinq participants (tous hommes), notamment trois chefs des FOSA et deux personnels des ONGs , ont eu connaissances des attaques qui se sont déroulés en dehors de la période d'étude. Les participants inclus dans l’étude étaient 34 hommes et 2 femmes, et inclus personnel des structures de santé, les autorités administratives, membres du conseil de santé communautaire, une structure de gouvernance locale qui fournit supervision d’établissement de santé et soutient les initiatives de santé dans la communauté (Tableau 1).

Tableau 1. Profil des participants dans les entretiens avec les informateurs clés

| **Profil des participants** | **Nombre des participants** |
| --- | --- |
| Chef de structure de santé | 14 |
| Personnel des organisations non-gouvernementales | 8 |
| Autorités sanitaires du district ou régionales | 5 |
| Personnel des structures de santé (exclus chefs de structures) | 4 |
| Autorités administratives | 3 |
| Membres de conseil de santé communautaire | 2 |

Les entretiens duraient de 35 minutes à 80 minutes (hors temps pour le consentement éclairé). Les entretiens réalisés ont porté sur 39 attaques différentes : 16 dans la Vakaga, 12 dans la Haute-Kotto et 11 dans la Ouaka. Le délai de ces attaques s'étend de février 2016 à décembre 2020. Sur les 39 attaques, 12 se sont produites dans des hôpitaux, 12 dans des centres de santé, 10 dans des postes de santé et 5 sur la route vers ou depuis une formation sanitaire.

### Contexte

Des groupes armés, y compris des milices locales et des milices originaires du Soudan et du Tchad, ont attaqué les soins de santé dans les trois préfectures étudiées dans un contexte de violence et d'insécurité permanente qui a entraîné des déplacements de population, une insécurité alimentaire, des problèmes d'eau et d'assainissement, des épidémies de maladies infectieuses, des obstacles financiers à l'accès aux soins de santé, une pénurie de ressources humaines et une méfiance à l'égard du système sanitaire. L'insécurité a également rendu les routes dangereuses, ce qui a compliqué l'accès aux soins et la fourniture de médicaments et d'équipements aux FOSA.

La population a énormément souffert. Un participant a déclaré :

*“L’étendu du dégât, je peux dire que c’est … je ne sais pas comment vous qualifier hein quand y’a des attaques y’a destructions comme ça vous imaginez l’impact sur la communauté, la communauté est déjà une [communauté] Centrafricaine qui n’a pas de moyens pour se prendre en charge on vient tout détruire encore détruit sa maison, bruler tout, tout il se retrouve avec tout ce qu’il a comme…. Mais il devient mendiant qu’est- ce qu’il peut si y’a pas des gens de bonnes fois pour l’appuyer mais il peut mourir de la malnutrition.”*

Les attaques contre les soins de santé étaient tellement omniprésentes que de nombreux participants ont eu du mal à faire la différence entre les attaques qui se sont produites pendant la période étudiée et celles qui se sont produites avant et après. Certains participants voulaient faire comprendre la taille et l'ampleur du conflit, tandis que d'autres avaient du mal à différencier les événements et l'impact des diverses attaques.

### Attaques Contre Les Formations Sanitaires

Les participants ont indiqué que les attaques contre les FOSA et leur personnel se produisaient aussi bien dans le cadre d'attaques plus larges contre les villages ou les villes et sur les routes, que dans les cas où la violence visait spécifiquement une formation sanitaire. Les prestataires de services ont parfois été averti de l'imminence d'une attaque par les habitants des villages voisins ou par d’autres passants. Le plus souvent, cependant, les participants ont rapporté que certaines attaques avaient eu lieu sans préavis, obligeant le personnel soignant, avec la population locale, à subir des violences ou à fuir avec peu de nourriture, de vêtements ou d'articles ménagers. Certains ont vécu dans les champs ou en brousses pendant des semaines ou des mois, en fonction de la position des attaquants et le contexte sécuritaire. Les prestataires ont fait preuve de bravoure et d'un jugement remarquable dans de nombreux cas. Dans un cas, en l'absence de moyens de transport propres, le prestataire a loué une moto pour évacuer la maternité et les services d'hospitalisation avant l'arrivée des assaillants. Un autre participant a raconté l'histoire d'une sage-femme de la FOSA qui a eu la clairvoyance de demander à d'autres personnes d'emporter des boîtes de médicaments pendant qu'elles fuyaient l'établissement. Ces boîtes ont ensuite été utilisées pour soigner la population en attendant que l'établissement soit réapprovisionné.

Selon les participants, les raisons perçues pour les attaques variaient : 1) Piller l’argent, les médicaments et les biens que les attaquants pourraient vendre ou utiliser ; 2) semer la peur dans la communauté ; 3) se venger de soins prétendument inadéquats prodigués à un des leurs (camarade) ; 4) obtenir des soins prioritaires pour les membres du groupe ; et 5) se venger contre le personnel de la FOSA d'avoir fourni des soins aux ennemis du groupe. Les groupes armés s’efforcent très souvent à causer plus de dégâts possibles, en utilisant des grenades, des armes à feu et parfois mettant le feu à la structure sanitaire, aux véhicules et au matériel.

Un participant a décrit un incident au cours duquel une bataille avait eu lieu près de la ville,

“*Un combattant étaient grièvement blessé sur le champ de bataille. Au moment où ces collègues-là amenées à FOSA, un médecin [était] en train d’opérait [un autre patient] au bloc opératoire. Ces collègues s’étaient surgis dans l’hôpital en exigeant de lui donner un soin de qualité, dans le cas contraire s’il décède, le médecin sera le responsable*“.

Les pillages pouvaient être importants, allant parfois jusqu'à la destruction complète de l'établissement. Les participants ont indiqué qu'un établissement de santé géré par une ONG était probablement plus vulnérable parce qu'il disposait de plus de ressources et pouvait générer plus de profits pour les attaquants. Une personne interrogée a expliqué :

*“Ce que s’est passé dans ses formations sanitaires dans un premier temps, les gens pensent qu’il y’a l’argent à l’hôpital, ils viennent en première position c’est pour chercher la caisse et rechercher à fouiller l’argent. S’ils ne trouvent pas l’argent c’est pour prendre des médicaments aller revendre et dans le cas contraire y’a pas de médicaments, c’est pour prendre certains matériels pour aller revendre. Dans les pires des cas s’ils ne trouvent pas quelque chose qui pouvant leur permettre d’avoir un peu d’argent pour subsister, ils brûlent les restes.”*

Un participant a indiqué qu'un groupe armé avait pillé une installation et détruit la maison de son directeur.

*“L’attaque qui était passé c’est ça que je vous ai dit, c’est une guerre ethnique entre les Rounga et les Kara, bon après [un affrontement entre les Rounga et les Kara], les Rounga ont fuient pour aller se réfugier au centre de santé … pour avoir des soins pour ceux qui étaient blessés et des enfants qui étaient malades….* *Quand [les Kara] étaient arrivés au sein de centre de santé, ils ont visé la maison du chef de centre. Ils ont pris les matériels de l’hôpital tel que, seringue, les médicaments et deux motos [et incendier le centre de santé]. [Ils ont] détruit carrément sa maison.”*

Souvent, après le pillage, les combattants détruisent le matériel et les médicaments qui ne les intéressent pas ou qu'ils ne peuvent pas emporter avec eux. Dans certains cas, le pillage s'accompagné d'un incendie criminel, du lancement d'explosifs, tels que des grenades, ou d'autres dommages causés à l'installation.

Les FOSA ont également fait l'objet de prises de contrôle par des groupes armés, soit pour s'en servir comme base militaire ou boucler stratégique, soit pour exiger des soins pour leurs malades et leurs blessés en contrepartie. Dans un cas, un participant a déclaré :

*“Le centre a été occupé par les hommes armés et c’était leur base. C’est de là qu’il allait commettre des gaffes dans le village."*

Les FOSA ont également été touchées lorsque des personnes déplacées à la suite d'une attaque y ont trouvé refuge. L'hébergement des personnes déplacées à l'intérieur du pays perturbe les services et augmente parfois les tensions et le risque d'attaques. Un participant a déclaré.

*“Les populations de [village X] et [village Y] se sont retranchées au niveau de l’hôpital, ils sont restés pendant deux à trois ans….A l’hôpital, la condition de vie de la population était très difficile ; ils n’avaient rien à manger ils n’étaient pas pris en charge, ils ont bénéficié de quelques soins après plusieurs mois d’inaction, certains trouvaient la mort par manque de soin c’était un désordre total, ils savent pas comment il faut faire pour survivre.”*

Les attaques répétées contre la même FOSA ont perturbé le rétablissement et affaibli davantage le système sanitaire. Les attaques menées dans les régions voisines ont entraîné des répercussions sur les établissements situés à proximité.

“*Ces attaques faisaient parties des attaques répétées et organisées mêmes, donc, ce ne sont pas des attaques sporadiques ou bien ciblées.*”

### Attaques contre les prestataires, les patients, et les familles

Les participants ont indiqué que les travailleurs de la santé étaient souvent confrontés à des risques élevés d'attaque en raison de la nature de leur travail. Comme démontré ci-dessus, ils travaillent avec des ressources telles que des médicaments, des moyens de transport, des liquidités et d'autres matériaux susceptibles d'attirer l'attention des groupes armés. Ils travaillent parfois dans des structures qui sont les mieux construites de la localité. Comme les personnels de la santé sont souvent obligés de se déplacer pour leur travail, de se réapprovisionner en intrants, de soumettre des rapports et de mener des activités de sensibilisation, ils sont vulnérables aux attaques sur les routes. De plus, ces derniers sont obligés de collaborer, de coopérer et surtout de soigner sans distinction.

Les participants ont rapporté que les groupes armés ont tué, violé, enlevé, battu et menacé des travailleurs de la santé et parfois leurs familles proches. Parfois, ils ont été attaqués au cours d’activités de soins ou lors du pillage de leur FOSA. Dans d’autres cas, ils ont été spécifiquement ciblés pour les mêmes raisons que les FOSA, par exemple l’insatisfaction quant à la qualité des soins, la fourniture de soins à des ennemis, la volonté de semer la peur et de déstabiliser les localités. Ils ont également été attaqués parce qu’ils étaient considérés comme riches ou parce qu’ils résistaient aux exigences d'un groupe armé.

Même si parfois des personnels de santé ont été tués sans planification pendant le chaos des pillages, souvent les participants ont estimé que les meurtres étaient intentionnels. Les meurtres ont visé spécifiquement les prestataires en guise des actes des représailles, sans faire de choix, tout en se déplaçant pour se réapprovisionner en intrants ou en cours des visites des sites pour des soins ou des vaccinations.

*“ Quand un des membres du groupe armé décède à la l’hôpital, ils accusent directement les agents de santé…. Ils étaient venus tuer le major de l’hôpital au lieu de son service…. Les groupes armés faisaient irruption dans les hôpitaux comme bon leur semble avec des grenades, des couteaux et des armes et menaçaient les agents de santé.*”

Certains membres du personnel ont été battus ou blessés et sont morts plus tard des suites de leurs blessures. Plusieurs participants ont indiqué que des collègues avaient été tués à leur domicile. Un participant a décrit comment un collègue avait survécu à une attaque mais avait été tellement battu qu'il continuait à souffrir de ses blessures au moment de l'entretien, plus de deux ans après l'attaque. Un participant a expliqué :

*“Il est tombé malheureusement sur les groupes armés qui l’ont tabassé et il a eu beaucoup de blessures. Jusqu’aujourd’hui, il continu de souffrir des séquelles de cette attaque. Il ne se porte pas encore bien.”*

Certains enlèvements ont été brefs, quelques heures, pendant que les groupes armés demandaient une rançon. D'autres ont duré des mois ou des années. Un participant a décrit l'enlèvement de cinq membres du personnel en 2019. Deux des cinq personnes enlevées sont toujours portées disparues, sans que l'on ait de nouvelles d'elles au moment de l'entretien de décembre 2022.

*“Ils avaient fait irruption dans la FOSA [et] séquestré un homme et quatre femmes... Quelques instants après, ils avaient relâché les deux vielles femmes qui nous ont relaté que l’homme qui a été séquestré a été fortement tabassé … Jusqu’aujourd’hui les deux autres femmes séquestrées ne sont pas encore relâchées et [les quatre femmes] avaient été violées."*

Le prestataire de santé a également été fréquemment menacé de violence. Un participant a expliqué :

*“Soit ce sont des menaces verbales qui remplis par des conditions de leurs formalités soit des châtiments corporels et même ça peut finir par la mort. Moi personnellement j’ai été victime d’une menace verbale mais le châtiment corporel non plus, je suis vraiment très docile.”*

Des menaces ont souvent été proférées à l'encontre du prestataire et des patients lorsqu'un groupe armé établissait un camp ou entrait dans une ville ou un village. Les menaces verbales étaient utilisées pour contraindre le prestataire de soins à engager des membres de groupes armés pour des activités sanitaires, telles que des campagnes de vaccination, ou pour obtenir des soins prioritaires dans les FOSA. Un participant s'est également souvenu d'une situation où un survivant d'une blessure par balle avait été amené à l'hôpital en ambulance, avait subi une intervention chirurgicale et avait été menacé par des chefs d'un autre groupe armé et par les soignants d'autres patients. Bien que le prestataire ait pu déplacer le patient dans une autre chambre d'hôpital pour le séparer des patients d'un autre groupe armé, et qu'il soit resté vigilant pour s'assurer que le patient n'était pas attaqué, le patient a quitté l'hôpital bien avant qu'il ne l'ait fait, parce que l'hôpital ne pouvait pas assurer sa sécurité.

Les membres de la famille des prestataires de soins de santé étaient également en danger. Lors d'une attaque, le directeur du centre de santé a été enlevé, ligoté et battu à mort. Un membre de la famille du prestataire a également été enlevé et assassiné, probablement en raison de son association avec le prestataire.

Des groupes armés ont également tué des patients. Une personne interrogée a déclaré :

*“ Ils ont fait irruption au centre de santé, tiré partout voire [même] sur les malades, mêmes ceux qui étaient sous sérum [perfusion].”*

Comme la population, les prestataires de soins ont perdu leur logement, leurs moyens de transport et leurs objets personnels lors des attaques, et ont été déplacés ou ont regagné leur domicile d'origine. L'un d'entre eux a déclaré :

*“[Après l’attaque] J’étais parti me réfugier [au site des déplacés] pendant 3 ans. Je suis retourné à la maison [cette année] et je dors dans la bâche.”*

### Impacts des Attaques

#### Impacts sur les FOSA et les Communautés.

Les participants ont indiqué que les attaques contre les FOSA ont interrompu les services pendant des périodes variées. Dans plusieurs cas, certains services de santé ont été fermés définitivement. Les participants ont indiqué que la population devait souvent chercher d'autres sources de soins, soit en parcourant de grandes distances pour atteindre les FOSA voisines, soit en se tournant vers des sources de soins traditionnelles. Un participant a déclaré que tout le village, y compris le personnel de santé et les bénévoles, avait fui pour le Soudan.

Des efforts considérables ont été déployés pour rouvrir les services peu après une attaque, mais les prestataires et les communautés ont dû faire face à des défis administratifs et opérationnels considérables. Les prestataires des FOSA se sont efforcés de réhabiliter les FOSA et de les débarrasser des débris. Les effets directs des attaques ont souvent été exacerbés par l'insécurité général**isée** dans la localité, ce qui a rendu difficile à transporter les matériaux de construction et réapprovisionner les FOSA. Certains participants ont indiqué qu'ils avaient besoin de l'approbation des hauts responsables de la santé pour rouvrir les services. Dans certains cas, la réticence à rouvrir était liée aux risques d'attaques ultérieures.

Lorsque les FOSA ont rouvert malgré les dommages causés par les attaques, ce qui pouvait se produire en quelques semaines pour les FOSA les plus accessibles ou en quelques années pour les plus inaccessibles, elles ont dû faire face à d'énormes défis. Les dommages subis par la structure pourraient empêcher le prestataire d'assurer un triage correct, un flux de patients ou même un abri de base contre les éléments pour les patients. Les participants ont parlé de toits tellement endommagés qu'ils ont été complètement arrachés.

En plus des dommages structurels, le personnel n'a souvent pas été en mesure de maintenir la qualité des soins en raison du manque de fournitures, d'équipements et des prestataires suffisamment formés. Parfois, un établissement rouvert fonctionnait avec des services temporairement réduits ou interrompait définitivement certains services. Les services interrompus étaient notamment ceux destinés aux groupes les plus vulnérables : maternité et santé génésique, prise en charge de la malnutrition aiguë, chirurgie et soins liés au VIH. Les personnes traitées pour la tuberculose et le VIH ont souvent manqué des traitements, ce qui risquerait d'accroître la résistance aux médicaments. À la suite d'une attaque, un établissement a suspendu ses activités de vaccination en dehors de la formation sanitaire (ex., à travers des stratégies avancées), ne fournissant les vaccins que sur place, ce qui a créé un obstacle supplémentaire à l'accès aux soins. Certains établissements ne se sont jamais complètement rétablis, fonctionnant avec des ressources plus limitées pendant des mois ou des années. Dans le pire des cas, les établissements ont fermé définitivement, laissant les communautés sans soins et surchargeant les établissements voisins.

Les participants ont décrit les conséquences des attaques, des déplacements et des restrictions de mouvement sur la santé physique et mentale des patients et leurs familles. Ces effets se sont souvent prolongés pendant de longues périodes et ont été exacerbés par la déstabilisation et parfois la dissolution de la communauté. Après les attaques, les communautés se sont retrouvées davantage exposées aux risques de décès et de maladies, que ce soit en raison des effets directs des attaques, tels que le déplacement, la mort ou les blessures, ou des conséquences indirectes telles que le traumatisme psychologique, la détérioration des relations sociaux, l'incapacité à travailler, et des conditions de vie précaires. Les dommages infligés au système de santé amplifient le risque que les problèmes de santé ne soient pas diagnostiqués ou traités précocement, voire qu'ils ne soient pas correctement pris en charge, engendrant ainsi une détérioration de la situation. Les individus les plus vulnérables étaient les personnes âgées, les jeunes enfants et les personnes vivant avec un handicap.

Un participant a déclaré :

*“Présentement où je vous parle, il n’y a pas de formation sanitaire à [la ville] et la population souffre. Les enfants ne sont pas vaccinés, risque d’épidémie et les femmes enceintes meurent souvent à la suite de l’accouchement non assisté à la maison.*”

La fréquentation des établissements de santé a souvent changé, même après leur réouverture. Dans certains cas, peu de faits ont été signalés aux établissements dans la période suivant immédiatement une attaque, car les gens craignaient de se déplacer pour se faire soigner, même sur de courtes distances. Un participant a déclaré :

“*Dans un premier temps quand [il] y’a les malades ne peuvent pas se déplacer, même les gens normaux [qui ne sont pas malades], ne peuvent pas se déplacer, même n’importe qui ne peut pas se déplacer, seuls les belligérants. Ça c’est une [raison] et[il] y’avait un souci à l’accessibilité*.*”*

Dans certains cas, après la diminution initiale de l'utilisation des services, il y a eu une recrudescence au fur et à mesure que la situation se stabilisée et que les déplacements devenaient plus faciles. Dans une communauté, au cours de la période qui a suivi l'attaque, les habitant ont refusé d'être hospitalisés ou de passer la nuit dans une formation sanitaire par crainte pour leur sécurité. Les habitants faisaient également davantage recours à la médecine traditionnelle pour soigner les malades et les blessés, soit en raison des risques liés au déplacement, soit la peur de chercher les soins de santé, soit parce qu'ils savaient que le centre de santé n’était pas fonctionnel ou sous-équipé après une attaque. Un autre participant a expliqué :

*“Après ces évènements, ils avaient eu trop peur d’amener leurs enfants à l’hôpital, ceux qui sont loin ont préféré traiter leurs enfants traditionnellement ce qui a fait qu’il y’a eu beaucoup de décès.*”

Les attaques contre les FOSA ont également contribué au déplacement des communautés. Les participants considéraient que les déplacements sont étroitement liés à une détérioration de l'état de santé due à des abris inadéquats, à des conditions d'hygiène insuffisantes, à l'insécurité alimentaire et à une vulnérabilité accrue au paludisme (en raison des nuits passées à l'extérieur et de l'absence de moustiquaires) et à d'autres maladies parasitaires. Un participant a déclaré :

*“Il y’a eu combat […] là-bas pour qu’ils puissent venir se réfugier chez nous, mais malheureusement les combats les ont poursuivis jusqu’ici, et c’est ça qui les a poussés à prendre une autre destination.... Ils avaient fui les combats et ils avaient encore prit une autre destination après l’attaque de notre FOSA.*”

Les participants ont fait état d'une augmentation de la mortalité, en partie à cause du retard dans la recherche de soins de santé. Souvent, les personnes ont attendu si longtemps avant d'amener un membre de leur famille ou un ami malade à la structure de santé que la personne n'était plus traitable. Dans ces cas-là, le prestataire ne pouvait rien faire d'autre que d'assurer le confort des personnes mourantes. L'un d'entre eux a dit :

*“La mortalité intra-hospitalière^[[1]](#footnote-1)^… quand les gens arrivent au stade tardif et que nous le personnel de santé on ne peut pas faire autrement c’est là que ça fini par la mort, et ça fini par la mort mais les gens se comptabilisent cette augmentation de la mortalité intra-hospitalière est normale c’est lié aux effets d’insécurité y’a tous ces corollaires.”*

Les participants ont également décrit les effets psychologiques des attaques généralisées et répétées contre les civils, y compris le meurtre de membres de la famille et d'amis, les passages à tabac, les viols, les tentatives de meurtre et d'autres actes perpétrés contre eux par des groupes armés. De nombreux membres de la communauté étaient en état de choc après une attaque, éprouvant des sentiments de peur, d'anxiété, de colère, de dépression et de détachement.

*“Il y a trop de conséquences et inconvénients, les gens sont sur leurs pieds, la peur gagne tout le monde, tu ne peux pas acheter quelques choses de valeur et garder, cela joue beaucoup sur la population, les gens ont peur ? Moi par exemple, je n’ai pas l’envi de faire des enfants, car en cas de quoi comment tu vas fuir, je suis obligé de faire l’espace de naissances, lors des évènements, il peut courir et marcher lui-même. Car, on fuit à tout moment. Les conséquences sont nombreuses.”*

#### Impacts sur les Prestataires

Comme les autres membres de la communauté, les travailleurs de la santé ont souffert physiquement et psychologiquement des attaques. Souvent, ils ont été chargés de soigner d'autres personnes, alors qu'eux-mêmes avaient besoin de se rétablir physiquement, mentalement et émotionnellement. L'impact psychologique est l'une des principales raisons pour lesquelles certains membres du personnel ont démissionné de leur poste et ont cherché du travail dans des localités plus stables, prenant le temps de se remettre de leurs expériences. Les prestataires qui sont restés en poste étaient surchargés et manquaient de ressources, ce qui a entraîné un épuisement professionnel et des erreurs cliniques potentielles. Lorsqu'ils sont restés, beaucoup ont eu le sentiment que leur travail n'était pas apprécié par leurs patients et qu'ils étaient même parfois menacés par eux.

L'un d'entre eux a déclaré :

*“Bon, toi qui aides tout le monde dans le village, les démunies, les pauvres, les groupes armés, tu les aides. Après quoi, ils viennent, ils te menacent vraiment est ce que cela te fera du bien ? C’est ce que moi je sens comme conséquence. Je suis tombé malade après cette attaque pour reprendre après.”*

Les prestataires de santé ont continué à être affectés par la menace constante d'attaques répétées. Leur détresse est encore exacerbée par la peur et la démoralisation dont ils sont témoins chez leurs collègues. L'un d'eux a déclaré :

*“On travaille dans les conditions difficiles du côté psychologique parce qu’on sait qu’à tout moment on peut être agressé et… ça influence...le climat du travail et l’attitude au niveau du travail…si les personnels se sentent menacer et y a des rumeurs que, voilà dans tels jours comme ça ils vont avoir des attaques ; vous voyez non ?* *C’est normal d’avoir peur et puis ça fait partie [des difficultés]… Donc ça joue nécessairement sur leurs patients et avec les stresses et les conditions de travail.”*

En outre, les travailleurs de la santé ont déclaré, avoir moins accès aux médicaments et aux fournitures qu'auparavant, travailler dans des bâtiments endommagés et même manquer de lits et d'autres fournitures. Ils ont déploré leur incapacité à fournir la qualité de soins pour laquelle ils ont été formés. Dans de nombreux cas, ils pouvaient poser un diagnostic clinique, mais ne disposaient pas d’outils appropriés pour le confirmer. Lorsqu'ils parvenaient à poser un diagnostic correct, ils ne disposaient souvent pas des médicaments nécessaires pour les traiter. Beaucoup ont exprimé un sentiment d'impuissance à améliorer la situation ou à fournir des soins appropriés à la population. Ceux qui occupaient des postes de supervision ou d'approvisionnement ne pouvaient pas effectuer leur travail en raison des obstacles liés aux déplacements.

*“Vous savez aussitôt après cet évènement, tout le monde s’était retiré dans les sites et peu de temps après lorsque les gens ont commencé à regagner leur domicile c’est là où y avait eu beaucoup de cas de maladie et du coup nous nous sommes confrontés au manque de médicaments. Même quand les patients arrivent à l’hôpital ils n’ont pas de quoi à s’asseoir, il n’y a même plus de lits pour faire coucher les malades. ”*

Bien que cela ait été mentionné moins fréquemment, certains prestataires sont restés bloqués dans l'établissement ou dans un village ou une ville dont ils n'étaient pas originaires, paracerque des risques d’être attaqué en cours de déplacement. Ils n'ont pas pu prendre leur congé pour rentrer chez eux et passer du temps avec leur famille et leurs amis. En conséquence, ils se sont souvent sentis isolés, ont eu le mal du pays et se sont sentis démoralisés en ne sachant pas quand est-ce qu’ils pourraient revoir leurs proches. Leur incapacité à prendre des congés a également conduit à l'épuisement professionnel et à ses symptômes associés tels que la dépression, la perte de motivation, le sentiment d'impuissance, l'isolement, le doute de soi et les changements d'humeur. L'un d'entre eux a expliqué :

*“Les dégâts étaient considérables, ça a duré. Même l’un des nôtres a voulu se rendre en province, on lui a fait du mal, le [collègue], lui aussi a eu des dégâts. Il se rendait à [Village X] accompagné d’un collaborateur venu de Bangui [en mission d’appui du niveau central]. En chemin, ils ont été interceptés par les groupes armés qui les ont dépouillés de toutes les motos et même des téléphones de service, tout a été emporté.”*

Dans de nombreux cas, les prestataires et les superviseurs ont évoqué la difficulté de trouver un équilibre entre la nécessité de fournir des soins de santé à la population et le risque encouru par les prestataires. Ils ont exprimé leur volonté d'apporter leur soutien, même dans un contexte aussi difficile, mais ont reconnu que s'ils étaient également blessés, ils seraient de toute façon incapables de s'occuper des patients. L'un d'eux a déclaré :

*“Les conséquences[sont que], on ne peut plus œuvrer là-bas parce que déjà c’est question de sécurité. Y avait un manque du personnel là-bas. Mais ce personnel venu qui est allé et amener là-bas devrait être un soulagement mais s’il est attaqué comme ça on ne peut pas continuer à prendre des risques de les amener dans les risques comme ça.”*

### Prévention et Atténuation

*Mesures Visant à Renforcer la Sécurité*

Les forces armées nationales et la Mission des Nations Unies en République Centrafricaine (MINUSCA) ont parfois aidé à sécuriser des zones après une attaque, mais n'ont principalement assuré la sécurité qu'à l'intérieur de leurs bases. Dans un cas, une base de la MINUSCA est devenue le centre de santé de facto jusqu'à ce que la situation se stabilise et que le centre de santé puisse rouvrir. Dans certains cas, les prestataires ont déplacé des objets volumineux et précieux, tels que des réfrigérateurs et des véhicules, vers les bases de la MINUSCA. Lorsqu'ils ont sécurisé une zone après une attaque, ils ont rassuré les habitants pour qu'ils rentrent chez eux et ont donné aux prestataires du centre de santé la confiance nécessaire pour rouvrir les services. La protection de la MINUSCA est limitée, non systématique et a souvent lieu des heures ou des jours après la fin d’une attaque.

L’un des participants a déclaré :

*“ [La fermeture de l’hôpital] n’a pas trop duré… à peu près deux semaines. Les Nations Unies étaient venues et on a rouvert la porte de l’hôpital.”*

La sécurité reste cependant un problème persistant et le personnel déplore souvent le manque de soutien qu'il reçoit. Une personne interrogée a déclaré :

“*Carrément, il n’y a personne. Personne ne pouvait venir ici, le problème c’est au moment où l’Etat était absent, et nous ne pouvons pas faire des mouvements, j’ai pu envoyer la nouvelle au niveau du district et ils n’ont rien dit. Même Bangui était informé. Carrément personne n’était ne pouvais venir, grâce à l’arrivée de l’IMC et elle était informée, mais elle ne pouvait rien faire. Si c’était l’Etat qui nous demandait comme tu le fais, on allait leur expliquer cela, mais carrément tout était fini comme ça, rien n’était, ils sont venus piller le village et le centre et ils ont repartis, il n’y avait personne pour en parler.*”

La prévention des attaques nécessite souvent un engagement avec les groupes armés et surtout une grande neutralité vis-à-vis des parties en conflits. Les membres de la communauté et les dirigeants locaux ont souvent réussi à négocier des accords avec les groupes. La volonté des prestataires de soins de traiter tous les patients s'est également avérée utile. Un participant a raconté qu'un membre de la communauté avait négocié un cessez-le-feu avec des membres d'un groupe armé en leur disant qu'ils devaient quitter l'hôpital en paix afin qu'ils puissent eux aussi bénéficier des soins prodigués dans cet établissement.

“*Ils n’ont pas touché au grand hôpital régional. Eux même ils se réjouissent du fait qu’ils sont aussi traités dans cet hôpital. C’était au début qu’ils allaient jusqu’à l’enceinte de l’hôpital à la recherche de certaines personnes afin de les enlever pour aller exécuter ailleurs. Mais c’est un pasteur qui les en avait empêché en disant qu’un hôpital est un lieu où on ne peut pas pénétrer dedans avec une arme à la main. Et c’est comme ça qu’ils ont renoncé à cela. Ils n’agissent plus comme auparavant.”*

Lors des campagnes de vaccination, ils ont souvent contraint les prestataires à les consulter pour obtenir un passage sûr ou même à engager leurs membres pour les aider. Si cette pratique compromet l'indépendance des services, elle assure également un certain niveau de sécurité aux prestataires. Un participant a déclaré :

*“Les campagnes de vaccination s’organisent d’une manière périodique, généralement quand il y a la campagne de vaccination on est obligé de rapprocher des responsables de ce groupe armés de mettre à notre disposition certaine de leurs éléments ou on loue leurs motos pour donner aux agents pour faire des activités avec. Donc c’est obligatoirement qu’ont les impliquent, si on les implique c’est à ce moment-là ils vont sécuriser les agents qui vont dans les ménages ou dans les villages pour vacciner les enfants.”*

Les cas d’engagement directe des groupes armés dans les activités sanitaires sont toutefois exceptionnels. Le plus souvent, les groupes armés se livrent à des actes d'extorsion et d'intimidation. Ils imposent des "impôts" aux véhicules sanitaires circulant sur la route. La présence de groupes armés dans un établissement, même pour un traitement, dissuadait les gens de se faire soigner et effrayait les patients. Un participant a déclaré :

*“C’est au courant du mois de février 2016 [le service chirurgical] n’a pas bien fonctionné. Tous les malades étaient aux aguets, puisque celui qui était blessé il avait passé 10 jours à l’hôpital et que ces collègues sont venus de temps à temps pour lui rendre visite, les gens ont peur.”*

Certains mécanismes utilisés pour protéger les communautés ont également aidé les FOSA. Par exemple, certains participants ont mentionné de plans d'évacuation, la résolution des tensions religieuses sous-jacentes alimentant les attaques, et de la défense armée des communautés, en particulier en ce qui concerne les groupes en provenance du Soudan et du Tchad. Un participant ayant une grande expérience de la région a décrit le désespoir des communautés à sauvegarder leurs FOSA. Lors des attaques, elles ont donné la priorité à la défense de ces FOSA par rapport à d'autres zones de la ville, reconnaissant que leur vie dépendait souvent des soins de santé qu'elles fournissaient. C'était particulièrement le cas lorsque la formation sanitaire avait été construite par des membres de la communauté.

*“La communauté a beaucoup souffert du manque de soins de santé. Chaque fois qu’ils disposent d’une formation sanitaire, Ils ont vraiment à cœur de le conserver. Ils défendront tout ce qu’ils ont. Ces formations sanitaires sont endommagées par une violence extrême lorsque la communauté ne peut plus les défendre.’’*

#### **Mitigation des impacts sur patients et la communauté**

Les membres de la communauté et les prestataires de soins ont fait preuve d'une grande persévérance face aux attaques. Les participants ont cité la mobilisation de la communauté grâce aux efforts des conseils de santé communautaire, des relais communautaires et des prestataires comme principale stratégie pour atténuer l'impact des attaques. Ces membres de la communauté ont utilisé leurs ressources personnelles et ont compromis leur sécurité et celle de leur famille, après avoir subi des menaces, des violences ou la perte d'un être cher ou de collègues, pour aider la communauté à se rétablir. Dans certains cas, les agents de santé se sont rendus dans la brousse pour fournir des soins aux personnes qui ne pouvaient pas se rendre dans les centres de santé.

*“C’est plutôt moi qui allais à sa rencontre dans la brousse pour les soigner. Surtout les petits enfants jusqu’à ce que tout le monde regagne le village.”*

Un participant a indiqué que les membres de la communauté avaient fait don de leur propre argent pour acheter une petite quantité de médicaments et de matériel en attendant que l'établissement soit réapprovisionné. Parfois, les établissements ont limité le traitement aux affections pour lesquelles ils disposaient de médicaments et plateau technique approprié. D'autres établissements ont continué à proposer des consultations et, lorsque les médicaments n'étaient pas disponibles, ont rédigé des ordonnances en espérant que les patients puissent les faire servir ailleurs et dans une grande majorité chez les vendeurs ambulants dans les kiosques au bord de la route. Pour les obtenir, cependant, les patients devaient payer de leur poche et devaient souvent s'adresser à des vendeurs non agréés et à des pharmacies privées. Un participant a expliqué :

“*Mais la majeure partie de la population a préféré se soigner sur place car il y a des commerçants qui vendaient beaucoup de médicaments ici sur place, on donne des ordonnances et les gens achètent des médicaments ici [dans la rue].*”

Les membres du personnel originaires de la communauté où ils travaillaient se sont adaptés plus rapidement après les attaques ; ceux qui venaient d'autres régions du pays (personnel qualifié) étaient plus enclins à partir. Lorsque les prestataires accrédités ont fui, les secouristes locaux et les agents de santé communautaires ont souvent pris le relais pour fournir des soins. Les prestataires restant leur ont dispensé une formation. Dans certains établissements, les services n'ont pas été interrompus grâce au dévouement du personnel local et des membres de la communauté. D'autres n'ont connu que des interruptions de courte durée, s'organisant de manière à pouvoir reprendre les services dès que possible. L'un d'entre eux a déclaré :

*“J’ai recruté un agent pour le remplacer et nous avons recruté une femme et formée pour mettre à la place de la sage-femme…C’est le secouriste que nous l’avons formé, il a pris la place du frère et l’assistant est monté à la place du major.”*

Les participants ont parlé avec force de leurs efforts de plaidoyer déterminés pour obtenir de l'aide, réhabiliter les FOSA et rétablir les services. Certains ont réussi, bénéficiant d'un bon soutien de la part des acteurs du système sanitaire au niveau local ou du district. Les infirmières en chef, les superviseurs, les responsables de la santé et d'autres ont répondu aux demandes des prestataires de voyager pour soutenir leurs collègues, de plaider pour remplacer les prestataires qui avait fui et de faire des demandes de stock auprès des ONG, des Nations Unies et du MSP. Ils ont également salué le dévouement et le courage de leurs collègues qui sont restés sur place et se sont donné beaucoup de mal pour continuer à prodiguer des soins. L'un d'entre eux a déclaré :

“*Moi, étant un chirurgien, je ne pouvais pas croiser les bras et regarder les femmes mourir devant moi, alors je suis évertué pour trouver ce qu’il fallait pour travailler. Et je suis descendu à [la ville] demander [à l’autorité sanitaire] de contacter les ONG médicales afin de nous venir en aide dans ce domaine car dans la commune… c’est le seul grand centre où tous les postes de santé de la commune y réfèrent les maladies graves. C’est pourquoi nous ne pouvons pas rester bras croisés et regarder les difficultés défilées devant nos yeux.”*

Les participants ont noté des ajustements informels des politiques et des pratiques. L'un de ces changements a consisté à passer de la fourniture de petites quantités de médicaments aux patients (contraire aux normes qualité de soins), ce qui garantissait un suivi régulier et une réduction dans la possibilité des ruptures des intrants, à l'augmentation des intrants fournit pour les permettre de tenir plus longtemps. Pour les personnes vivant avec le VIH, le MSP a commencé à dire aux prestataires de donner suffisamment de médicaments antirétroviraux pour une durée de 5 à 6 mois afin de limiter les visites des patients, de réduire leurs déplacements et leur exposition aux risques aux attaques des groupes armés. Les prestataires ont fait de même pour les patients inscrits à des programmes d'alimentation thérapeutique ambulatoire pour la malnutrition, en leur donnant des aliments thérapeutiques prêts à l'emploi pour un mois ou plus, au lieu des deux semaines habituelles. Cet ajustement a également permis de garantir que les clients et les patients pourraient être soignés eux-mêmes au cas où les stocks des produits arrivaient à être pillés. Pour des raisons similaires, les prestataires ont utilisé une stratégie décentralisée, en fournissant aux centres de santé du matériel qu'il n'aurait normalement fourni qu'aux structures de référence. Certains centres de santé ont reçu des kits d'urgence pour pratiquer des césariennes, alors que la politique habituelle exigeait que les accouchements compliqués soient référés.

*“Pendant cette période le cas de référence a beaucoup diminué…. Même le cas de malnutrition ne venait pas, les cas de paludisme ne venaient pas aussi. Je pense que [le staff] ont gérés ça à leur niveau. Après cet incident je n’ai écouté que [secouriste] à opérer deux cas de césarienne des femmes enceintes et y à pas de problème. Il a pris la décision de faire parce qu’y avait un grand risque [d’attaque] encore. Parce que des patients qui n’ont pas été référé, c’est à cause de cette attaque.”*

Les responsables des centres de santé cherchant à minimiser l'exposition à la violence ont réduit l’effectif des prestataires en poste, bien que cela puisse compromettre la qualité des soins. Un participant a fait remarquer que si les zones rurales ont besoin de personnel qualifié comme les médecins, la perte de ce type de personnel hautement qualifié à cause d’une attaque épuise non seulement le système sanitaire déjà fragilisé, mais réduise aussi sa capacité à former d'autres personnes. Le MSP a parfois rappelé des prestataires qualifiés à la suite d'attaques contre les soins de santé. Afin de préserver leur sécurité, il a également demandé à la communauté de s'assurer que la zone soit sûre avant d'envoyer du personnel hautement qualifié.

Les chefs de centres de santé accompagnés les patients dans des structures de références pour bénéficier de services qui n'étaient plus disponibles dans leurs propres formations en raison des effets des attaques, les amenant parfois dans leurs véhicules personnels. Les responsables ont préféré garder dans la FOSA un petit stock de fournitures et de médicaments dans les établissements et ont retiré l'argent liquide qui s'y trouvait. Cette stratégie a permis de réduire le risque de perte de biens en cas d'attaque, mais a augmenté le risque de rupture de stock, en particulier lorsque les déplacements étaient difficiles en raison de l'insécurité ou du mauvais état des routes. Parfois, s'ils savaient qu'un groupe armé allait passer par là, ils auraient envoyé des membres de la communauté cacher des provisions dans un endroit plus sûr. Les grands hôpitaux de la région ont parfois conservé des fournitures pour les établissements plus petits, les réapprovisionnant plus fréquemment avec de plus petites quantités, ou ont fourni des services qui ne pouvaient plus être assurés localement. Un participant a déclaré :

*“[Les enfants] reçoivent normalement leurs vaccins car l’hôpital envoi des personnels à formation sanitaire 2 à 3 jours pour les vaccinés et ensuite ramènent.”*

Les ONG internationales et les agences des Nations Unies, notamment IMC, Médecins Sans Frontières, Save the Children, l'Organisation Mondiale de la Santé (OMS) et le Programme des Nations Unies Pour le Développement (PNUD), ont apporté soutient aux FOSA après une attaque, en remplaçant souvent les médicaments et matériels sanitaires volés.

*“Après ce qui est arrivé, nous avons fait recours à l’un de nos partenaires à savoir l’IMC. On a échangé avec lui et c’est lui qui a doté le centre de santé de [FOSA X] en médicaments pour aider la population.”*

Les ONG locales ont également soutenu les structures de santé après une attaque, en particulier lorsque la population avait fui la zone et vivait dans des sites pour personnes déplacées. Dans certains cas, les ONG locales et internationales ont repris les services ou offert des soins directs, par exemple par le biais de cliniques mobiles, y compris dans les lieux où vivaient les personnes déplacées, et même dans la brousse.

Ces efforts ont été limités, car les ONG internationales et locales ont été confrontées aux mêmes problèmes de sécurité et de déplacement que la communauté et les prestataires du centre de santé. Dans certains cas, après une attaque, les ONG ont suspendu leur aide ou se sont carrément retirées de la localité, ce qui a plongé la communauté dans un sentiment de désespoir et d'impuissance. Certains professionnels de la santé qui ne pouvaient plus travailler en toute sécurité dans leurs FOSA ont déménagé dans d'autres lieux. Un membre du personnel d'une ONG a indiqué qu'il comptait beaucoup sur la communauté pour négocier avec les groupes armés et assurer la sécurité du personnel. Certains participants ont également nommé la portée limitée des bailleurs, qui n'approuvent l'aide que pour courtes périodes pour des services limités, comme un défi à relever dans les zones d'insécurité.

Les participants ont également un avis mixte sur les mesures gouvernementales d'atténuation et de soutien. Une personne interrogée a crédité le Ministère du Plan d'avoir signé des accords avec des ONG internationales et nationales pour fournir des services directs sur ces sites. Un autre a mentionné la réticence de MSP à renvoyer des prestataires accrédités dans des zones peu sûres à la suite d'une attaque. D'autres, en revanche, ont déclaré avoir reçu peu de soutien de la part du gouvernement centrafricain, notamment du MSP et de ses autorités sanitaires régionales et de district, en ce qui concerne le réapprovisionnement, la réparation des FOSA et le remplacement du personnel qui a fui. Ils ont déclaré avoir reçu peu d'informations de la part du MSP ou des agences concernant l'aide au rétablissement des services, et que personne n'était venu ou n'avait appelé pour s'enquérir des détails de l'attaque.

*“Carrément, sinon, aucun agent n’est venu, aucune ONG, ni aucun personnel de l’Etat pour venir poser des questions sur les évènements. Nous, les enfants du village, sommes concertés pour maitrise la situation et voir ce qu’il y’a lieu à faire, mais il n’y avait aucune présence de l’Etat, même aucun projet, il n’y avait aucun suivi après l’attaque.*”

En partie, ces problèmes étaient le produit de difficultés de communication et de rapportage. De nombreux participants ne savaient pas très bien dans quelle mesure le gouvernement ou les Nations Unies avaient été informés de l'existence d'une attaque, ni quels en étaient les détails. Les mauvaises connexions téléphoniques et Internet, ainsi que la difficulté et l'insécurité des déplacements, ont rendu la déclaration difficile. Il a parfois été demandé aux voyageurs de partager l'information au niveau central, mais dans ce cas, de nombreux détails de l'incident ont probablement été perdus.

Rapportage était aussi limité dû à peur de représailles par les groupes armés et les participants ont rapportés que souvent il ne valait pas la peine de courir le risque de représailles, surtout s'ils n'étaient pas convaincus que le rapportage pourrait améliorer la sécurité. Les administrateurs principaux dans les provinces ont parlé des difficultés qu'ils rencontraient pour obtenir des informations sur les attaques. Lorsqu'une attaque est signalée, nombreux sont ceux qui informent leurs supérieurs hiérarchiques au sein du MSP. Plus rarement, les attaques étaient signalées à la MINUSCA.

## Discussion

Nos recherches en RCA révèlent un paysage complexe et alarmant d'attaques contre les formations sanitaires, leur personnel et leurs patients. Elles soulignent les impacts profonds et durables de cette violence sur la prestation des soins de santé et le bien-être de la communauté. L'omniprésence des attaques n'a pas seulement infligé des dommages directs, mais a également entraîné de graves perturbations opérationnelles, avec des fermetures prolongées ou permanentes de services, des pénuries criantes de personnel et des fournitures médicales insuffisantes. Les suites des attaques montrent un système sanitaire qui s'efforce de répondre aux besoins de la population au milieu d'un conflit permanent.

Les attaquants ont pillé des fournitures médicales, de l'argent liquide et des médicaments, endommagé, incendié ou détruit d'une autre manière des installations et menacé, tué, enlevé et agressé physiquement ou sexuellement le personnel. Certaines attaques semblaient faire partie d'une vague de violence infligée à une communauté, tandis que d'autres visaient spécifiquement les établissements et les prestataires. Les raisons perçues de la violence étaient variées : gain financier provenant de l'argent liquide ou de la vente potentielle de biens volés ; utilisation personnelle d'articles pillés ; colère des combattants face à la « mauvaise qualité » présumée des soins ; refus d'accorder la priorité au traitement des combattants ; représailles pour avoir traité leurs ennemis ; ou la FOSA est située dans une localité ciblée par une attaque plus large contre la population locale.

Les actes de violence contre les soins de santé dans les trois préfectures de l’étude (Haute-Kotto, Ouaka et Vakaga) correspondent aux conclusions des études menées dans d'autres pays en guerre, bien qu'il y ait des différences notables par rapport aux pays à revenu intermédiaire(20). À quelques exceptions près, le pillage est plus fréquent dans les conflits internes chroniques dans les pays à faible revenu d'Afrique notamment, au Burkina Faso, en République démocratique du Congo, au Mali, au Nigeria, au Sud-Soudan et au Soudan(20), ainsi que la République Centrafricaine.

Dans les pays à revenu intermédiaire, les systèmes sanitaires fonctionnaient raisonnablement bien et disposaient des prestataires en nombre suffisant avant la guerre. Les conséquences systématiques des attaques dans ces pays varient. Plus de 600 attaques contre des hôpitaux en dix ans ont gravement ébranlé le système sanitaire dans le nord-ouest de la Syrie(21). En Ukraine, en revanche, malgré plus de 1 000 attaques russes contre les soins de santé et le départ de nombreux membres des prestataires, le système sanitaire a conservé une fonctionnalité relativement élevée(10). En République Centrafricaine, comme dans d'autres pays à faible revenu, les conditions qui prévalaient avant le conflit, notamment l'extrême pauvreté, la faiblesse de l'économie, l'insuffisance du nombre de prestataires de santé, le mauvais état des infrastructures et l'héritage du colonialisme et des conflits passés, ont amplifié l'impact de la violence contre les soins de santé et entravé la capacité à s'en remettre(15, 22-25).

### Impact des Attaques

En raison des pillages, des dégâts matériels et de l’insuffisance des prestataires résultant des attaques sur les soins de la santé, les établissements ont souvent été confrontés à des retards de réouverture prolongés, parfois de plusieurs mois ou années ; certains n'ont jamais réussi à rouvrir. Pour ceux qui ont continué à fonctionner, le remplacement des stocks a été difficile en raison des dangers liés aux déplacements et du risque d'attaques futures. Conformément à d'autres rapports d’étude élaborés sur la RCA(26), celle-ci a observé que les FOSA ou leurs enceintes étaient devenues des lieux de refuge pour les personnes fuyant la violence, mettant à rude épreuve leur capacité opérationnelle, leur sécurité et augmente les problèmes d’hygiène et entretien des locaux. Reconstruire les infrastructures demande du temps et des investissements qui manquaient aux préfectures. En 2019, 18,6%, 36,0% et 5,0% des installations ont été partiellement détruites respectivement à la Ouaka, Haute-Kotto et Vakaga(27). En outre, 18,6% et 8,0% des installations ont été entièrement détruites en Haute-Kotto et Vakaga. Notre étude a indiqué que les défis étaient les plus grands pour les postes et centres de santé situés dans les zones les plus reculées des préfectures.

L’absence ou l’insuffisance des services de santé compromet gravement la santé de la population, exposant les individus à des risques accrus de maladie et de décès. Sans accès fiable aux soins médicaux, les maladies infectieuses peuvent s’aggraver de manière incontrôlée, des affections évitables ou facilement traitables se transformer en cas compliqués, des affections chroniques peuvent s’aggraver et devenir ingérables, et les femmes peuvent mourir inutilement en couches. Garantir des infrastructures et des services de santé solides est essentiel pour protéger la santé publique et réduire le risque de morbidité et de mortalité évitables au sein d’une population.

Les conséquences des fermetures prolongées, du manque de prestataires et des ruptures de médicaments et de fournitures sur les populations ont touché de manière disproportionnée les groupes les plus vulnérables. La qualité des services de santé pour les enfants, aux personnes âgées et aux patients ayant besoin de médicaments réguliers, comme les personnes avec tuberculeux et les personnes vivant avec le VIH et le sida, ont souffert. Certaines FOSA n'ont eu d'autre choix que de fermer les services de santé reproductive, de malnutrition, VIH et de chirurgie. Les vaccinations ont été interrompues, ce qui a contribué semblablement à l'apparition de maladies infectieuses(28). Ces obstacles aux soins ont probablement exacerbé les inégalités et les injustices existantes, affectant de manière disproportionnée les personnes vivantes plus loin des centres de soins et celles disposant de ressources économiques limitées. L’étendu du pays, la faible densité de la population, le pouvoir d’achat limité et le faible niveau d’instruction ont facilité les conséquences négatives dans la population.

En RCA, avant le récent conflit, de nombreux établissements manquaient de personnel possédant des qualifications professionnelles. De plus, il était extrêmement difficile de remplacer le personnel qui avait fui après les attaques, un problème amplifié par les difficultés préexistantes liées au recrutement et à la fidélisation du personnel dans les programmes de santé éloignés. La peur était un combat constant pour les travailleurs de la santé et les communautés, car ils ne savaient pas quand les attaquants frapperaient à nouveau. En 2019, dans les trois préfectures de l’étude, il n’y avait que 35 infirmiers, 11 médecins et 11 sage-femmes(27). La RCA compte 1,0 personnel médical qualifié pour 1 000 habitants, bien en dessous de la densité régionale africaine de 1,5 personnel pour 1 000 habitants(29) et l'OMS a recommandé 2,5 personnels nécessaires pour assurer une couverture adéquate des soins de santé primaires(30). Le recrutement de sage-femmes a peut-être été particulièrement difficile en raison du risque combiné d’agression sexuelle pour les femmes et de l’important écart d’égalité entre les sexes, ce qui a dégradé le statut des femmes dans la société(31).

Pour les travailleurs de la santé, le traumatisme psychologique lié à l'exposition aux attaques a été grave et durable. Comme dans d'autres études, le traumatisme a été aggravé dans de nombreux cas par le fait d'avoir vécu plusieurs attaques(12, 15, 16, 32). Certains membres du personnel ont été profondément affectés par le traumatisme psychologique de leurs collègues. En outre, comme en Syrie, ils ont subi un préjudice moral du fait qu'ils n'étaient pas en mesure de fournir les soins pour lesquels ils avaient été formés et se sentaient tenus de le faire, et qu'ils se sentaient impuissants à changer leur situation(12, 33).

### Stratégies de Mitigation

Pour atténuer les difficultés, les prestataires et le MSP ont eu recours à des stratégies de mitigation qui impliquaient des compromis douloureux. Le MSP a permis l’administration à domicile étendue de traitements tels que les antirétroviraux contre le VIH et les aliments thérapeutiques contre la malnutrition. La stratégie a atténué le risque de perte de stock lorsque les moyens de transport ou les installations étaient attaqués, mais a accru les risques d'une adhésion incohérente, augmentant ainsi la morbidité et la mortalité. Pour des maladies comme le VIH et la tuberculose, une observance incohérente augmente le risque de résistance aux antimicrobiens et aux médicaments. Les FOSA qui se sont adaptées en minimisant les stocks disponibles et les liquidités et en les stockant dans des endroits plus sécurisés ont réduit le risque de perte mais augmenté le potentiel de rupture de stock. La réduction du personnel en poste dans un établissement a protégé leur sécurité, mais a probablement compromis la qualité des soins et augmenté le risque d'épuisement professionnel.

Le MSP a hésité à renvoyer du personnel accrédité dans des zones non sécurisées après une attaque, craignant que le pays ne perde un membre du petit nombre de personnel médical formé. Cependant, leur absence entravait non seulement la capacité de diagnostiquer et de traiter les patients, mais excluait également la possibilité de former d’autres personnes. Les ONG internationales ont fréquemment réapprovisionné les FOSA à la suite des attaques et ont renforcé leurs effectifs. Certains d’entre eux ont créé des cliniques mobiles. En collaboration avec la Banque Mondiale, le MSP a tenté de réapprovisionner les médicaments et de remédier aux pénuries de personnel. Sa capacité à fournir un soutien a été entravée par des problèmes de communication, des difficultés d'accès aux installations touchées, un manque de ressources et souvent par un manque de connaissances sur la nature des attaques. Des difficultés persistaient dans les zones touchées par le conflit. Tout au long de la crise, IMC a été le seul partenaire à ne jamais quitter la préfecture de la Vakaga.

### Réponse de la Communauté

Les communautés, en collaboration avec les agents de santé qui en faisaient partie, ont fourni l'essentiel du soutien aux FOSA après les attaques. Les membres de la communauté ont comblé les lacunes en matière de prestataires après avoir eux-mêmes subi des violences et des menaces, au péril de leur vie et de celle de leur famille. Les prestataires recrutés localement était dite d’être plus susceptible de rester après une attaque et de chercher à soutenir l'établissement, tandis que les travailleurs non locaux partaient souvent. Lorsque les agents sanitaires ont fui, les FOSA ont fonctionné avec des agents de santé communautaires, des accoucheuses traditionnelles ou des personnes ayant reçu une formation aux premiers secours. Certains ont été formés par du prestataire accrédité, mais la qualité des soins en a inévitablement souffert. Les agents de santé restés après les attaques ont étendu leurs services aux endroits où les personnes déplacées avaient cherché refuge.

Dans certains cas, la communauté a renforcé la sécurité des installations en s'armant. D’autres fois, ils négociaient avec des acteurs armés. L'engagement direct avec les groupes armés est de plus en plus reconnu comme une stratégie clé pour améliorer la sécurité des programmes de santé. Cet engagement mérite une délicatesse compte tenu du caractère volatile des promesses faites. Certaines organisations internationales, dont le Comité international de la Croix-Rouge, la Croix Rouge Centrafricaine et MSF, travaillent avec les communautés pour favoriser l'engagement avec les acteurs armés. La volonté des ONG de s'engager auprès des groupes armés dépend souvent du contexte, des compétences et des relations avec les gouvernements nationaux(34). De nombreuses communautés dans les trois préfectures se sont engagées avec les groupes armés, malgré les risques, et sans la possibilité d’évacuation que les ONGs ont. Nos résultats sont cohérents avec une étude de Barbelet et al. qui a montré que les communautés en RCA réussissent souvent à s'engager avec les groupes armés, en particulier lorsque les groupes sont décentralisés et qu'ils ont des relations existantes au sein de la communauté(35).

### MSP et la Réponse Internationale

Le MSP a institué un plan quinquennal 2022-2026 de renforcement du système sanitaire, soutenu par la Banque Mondiale(36). Ce plan est en partie conçu pour atténuer les insuffisances d’effectif de prestataires de santé dans les régions de la RCA touchées par le conflit. Le plan prévoit des compléments de salaire pour les prestataires travaillant dans les zones mal desservies et des formations pour améliorer les compétences des travailleurs de la santé. Certaines ONG internationales et certains donateurs soutiennent ces initiatives, allant même, dans un cas, jusqu'à créer une école de formation pour les prestataires de santé en dehors de la capitale. Les résultats du suivi du projet ont indiqué que la stratégie est prometteuse pour l'avenir du système de santé(37). Bien que lancée après la période de l'étude, la stratégie représente un engagement important du MSP à soutenir les FOSA et les prestataires exposés à des risques d'attaques ou touchés par celles-ci, et s'est engagée directement auprès des groupes armés(38).

Les bailleurs de fonds internationaux ont réduit leur investissement dans le système sanitaire ces dernières années et leurs engagements envers la RCA ont diminué davantage après l'échec de la négociation d'un accord de paix en 2019 et l’implication du Groupe Wagner(39-41). La mauvaise gouvernance, et le manque d'impact des financements précédents peuvent également avoir conduit certains donateurs à réduire ou à suspendre leur soutien(37, 42). Dans les deux années qui ont suivi, le montant de l'aide à la RCA a chuté de 24 millions de dollars(43). En 2023, le montant du financement des donateurs pour la santé ne représentait que 60 % du montant demandé par le groupe sectoriel de la santé. Malgré son extrême pauvreté, la RCA se classe aujourd'hui au 54e rang mondial pour la réception de l'aide au développement(44). En outre, en 2023, l'aide en espèces et sous forme de bons aux particuliers a diminué de 17% par rapport à 2022, ce qui s'explique en partie par la baisse générale de l'aide à la République Centrafricaine(45). Il faut noter que les subventions accordées à la RCA, très peu a été utilisée au profit de la population centrafricaine et il y’a moins d’intervention qui s’inscrivent dans la durabilité(37, 42).

La MINUSCA, qui a un mandat fort de protection des civils, a joué un rôle modeste et disproportionné en matière de sécurité. Dans certaines situations et devant l’attaque des hommes armés, la MINUSCA est restée inerte sans protéger ces populations devant le danger. Les participants ont indiqué que la MINUSCA autorisait les prestataires à stocker du matériel et des fournitures dans leurs bases et, dans un cas, à y fournir des services. Cependant, à part quelques exceptions comme la présence d'un poste de contrôle à l'entrée d'un hôpital, la MINUSCA n'a pas assuré directement la sécurité des FOSA. Le rôle de la MINUSCA en matière de sécurité peut également être compliqué par la composition religieuse ou ethnique de la force(38). Cela a été considéré comme un facteur favorisant la partialité en réponse à une crise liée aux tensions religieuses et ethniques existantes.

### Rapportage et Persécution des Attaques

La notification et l'analyse des attaques, essentielles pour améliorer la sécurité, restent insuffisantes. La collecte de données est sporadique, les rapports ne sont pas diffusés aux parties prenantes et les informations recueillies ne permettent pas d'élaborer des stratégies visant à renforcer la sécurité sanitaire et à atténuer les effets néfastes des attaques. Le système de surveillance de l'OMS pour les attaques contre les soins de santé (SSA) souffre de défauts structurels et opérationnels qui l'empêchent de remplir son mandat, à savoir documenter les attaques(46). La majorité des cadres du MSP ne connaisse pas l’existence de ce dispositif. Sa dépendance à l'égard des données du groupe sectoriel de la santé, qui ne sont pas totalement représentatives, limite sa portée. Il ne partage pas les informations avec les autorités nationales, les communautés ou les ONG locales et ne fournit pas aux décideurs des détails tels que le lieu, le moment et la nature des incidents(47). Sa conception n'a pas été adaptée aux défis uniques de la déclaration en République Centrafricaine et dans d'autres pays en proie à un conflit prolongé. En outre, comme les données ne sont pas utilisées pour la protection ou le soutien, les prestataires de soins de santé sont peu incités à faire des déclarations. Au cours de la période d'étude 2016-2020, l'OMS a enregistré 82 attaques, 5 décès et 13 blessures dans 20 préfectures de RCA, par rapport aux 126 attaques que nous avons identifiées dans les trois préfectures de notre zone d'étude(46).

Les attaques contre les soins de santé dans le monde entier sont restées impunies, et les crimes de guerre commis contre les FOSA, les prestataires et les patients n'ont fait l'objet d'aucune poursuite(38). La Cour Pénale Internationale, qui a compétence sur les crimes de guerre et les crimes contre l'humanité en RCA depuis 2012, a déclaré en 2022 qu'elle n'ouvrirait pas d'autres enquêtes au-delà des affaires en cours, dont aucune n'impliquait d'attaques contre les soins de santé(48). En revanche, le procureur a mis l'accent sur la coopération avec la Cour Pénale Spéciale créée en 2015 pour enquêter sur les violations graves des droits de l'homme et des conventions de Genève en République Centrafricaine et poursuivre les auteurs de ces violations à partir de 2023. Ce tribunal souffre cependant de lenteurs administratives, de difficultés liées à la constitution des preuves. Ces éléments contribuent à la disparition des preuves et rendent les poursuites difficiles faute de preuves(49).

La Cour, en coopération avec les Nations Unies, comprend des juges, des procureurs et des administrateurs internationaux et nationaux. Bien qu'elle soit confrontée à des défis opérationnels en matière de prestataires, d'arrestation des auteurs de crimes, de protection des témoins, de financement et de sécurité, la Cour n'a pas été en mesure de faire face à ces défis, le tribunal a progressé en rendant son premier verdict pour un massacre de 46 civils en novembre 2022(50). En septembre 2023, elle a inculpé un ancien chef d'un important groupe armé, le Front populaire pour la renaissance de la République Centrafricaine, pour crimes de guerre et crimes contre l'humanité(51). Les accusations ne concernaient pas les attaques contre les soins de santé et de nombreux autres acteurs, qui ont directement et indirectement contribué aux violations des droits humains, restent en liberté.

### Une voie à suivre

Il existe des stratégies efficaces pour protéger les soins de santé de la population centrafricaine. Pour protéger les soins de santé de la violence et aider les communautés et les prestataires de soins qui y sont confrontés, un certain nombre de mesures essentielles doivent être prises. Le Gouvernement centrafricain et la communauté internationale doivent accroître leur soutien en évaluant les besoins, en renforçant la sécurité, en facilitant la communication et en atténuant l'impact des attaques par la reconstruction rapide, le réapprovisionnement et la réaffectation des FOSA. La communauté internationale doit cesser de se dérober à ses responsabilités en fournissant l'aide financière et les autres formes de soutien nécessaires aux soins de santé en RCA. La formule bénéfique doit s’inscrire dans la durabilité afin de faire le tremplin entre l’humanitaire et le développement, seul capable de donner une capacité résiliente au système de santé. Cela inclut des ressources pour la formation continue, le soutien psychosocial des travailleurs de la santé et des communautés qui ont été blessés et qui sont toujours en première ligne. Comme l'ont conclu Barbelet et al., les donateurs devraient investir dans les organisations communautaires et les groupes de la société civile afin de renforcer la capacité des communautés à prévenir la violence et à améliorer la résilience face à celle-ci(35, 52).

En outre, compte tenu des engagements déclarés du MSP, les donateurs autres que la Banque Mondiale devrait accorder la priorité au soutien du plan quinquennal, notamment en finançant la formation, les salaires des prestataires de santé et l’amélioration du plateau technique. Il est crucial de financer le remplacement des prestataires lorsqu'il est contraint de fuir la violence, et assurer la protection du personnel de première ligne, car le système sanitaire n'existe pas sans lui. Les donateurs devraient en outre veiller à ce que les prestataires perçoivent une rémunération adéquate et un soutien psychosocial approprié. Le soutien inconstant des donateurs, comme en témoigne la récente réduction ou la pause du financement, ne fera qu'exacerber les souffrances, perpétuer l'impunité et négliger les opportunités d'atténuer les conséquences de la violence. Bien que la mauvaise gouvernance ait été citée à chaque fois comme cause de faible performance du système de santé, très peu sinon aucun programme proposé par les acteurs mette l’action sur la mise en place d’un système efficace, efficient et redevable en vue d’endiguer de façon significative ces maux qui gangrènent le système de santé centrafricain.

La collecte de données devrait être explicitement liée à la protection, l'atténuation et surtout la mitigation des risques. L'OMS devrait remanier le SSA pour garantir son efficacité en RCA, en intégrant de nouvelles sources de données, en améliorant le partage des données avec les communautés et les parties prenantes, en renforçant la transparence des rapports et en collaborant efficacement avec le MSP, les ONG et les communautés pour élaborer des stratégies visant à atténuer l'impact des attaques.

Compte tenu des effets profonds de la violence contre les soins de santé sur leur disponibilité pour les populations et sur les victimes individuelles, le Tribunal spécial devrait donner la priorité à l'enquête et à la poursuite des crimes impliquant des violences infligées aux soins de santé. La Cour pénale internationale devrait en outre offrir un soutien vigoureux à la Cour pénale spéciale en RCA.

### Limites

Cette étude s'est concentrée sur les zones de captage dans trois préfectures de la RCA, largement soutenues par des ONG internationales, et peut ne pas représenter les conditions dans les autres zones soutenues ou non. L'étude a probablement obtenu des informations sur les attaques les plus graves. Compte tenu de la fréquence des attaques, du délai entre les attaques, la capacité de rétention et la collecte des données, les récits des personnes interrogées sont fortement sujets à des biais de mémorisation. La nature traumatisante et les effets psychologiques de la violence peuvent également avoir eu un impact sur la mémoire des événements par les participants. Malgré les efforts de triangulation des données avec d'autres sources, certains détails des attaques n'ont pas pu être corroborés.

De nombreuses personnes interrogées étaient réticentes à partager des informations par crainte de représailles. Les menaces étant très courantes, elles n'ont souvent pas été mentionnées et l'étude n'a donc pas pu distinguer les effets de menaces spécifiques.

L'étude s'est appuyée sur des entretiens avec des informateurs clés jouant un rôle public ou employés par des ONG, à l'exclusion des membres de la communauté n'occupant pas de postes de responsabilité dans le domaine de la santé ou de l'administration. Par conséquent, l'étude examine principalement les effets sur le système sanitaire et la prestation de services, les impacts sur les patients et les communautés étant abordés indirectement. Pour la même raison, nous n'avons pas recueilli d'informations sur la violence et les obstacles, tels que les barrières routières, qui ont empêché l’accès des personnes aux établissements de soins, sauf s’ils sont connus des informateurs clés.

Réaliser des entretiens par téléphone et Skype/Zoom présentait ses propres défis. Certains entretiens ont été affectés par une mauvaise connexion téléphonique ou Internet ; en résultats, les enquêteurs et les personnes interrogées devant se répéter ou entraînant des interruptions d'appels. Cela a probablement entraîné une certaine frustration de la part des participants à l'étude, et peut avoir conduit les participants à ne pas approfondir autant la discussion sur les attaques, ou à ne pas rapporter certaines attaques dont ils se souvenaient, car ils voulaient simplement conclure les entretiens.

Compte tenu de la nature sensible des entretiens, la positionnalité des enquêteurs aurait pu affecter les résultats. Les entretiens ont été principalement réalisés par un chercheur de l'ICASEES. La localisation éloignée de ces chercheurs, qui se trouvaient tous à Bangui, des informateurs clés situés dans les trois préfectures, a probablement réduit les inquiétudes potentielles des informateurs clés concernant la positionnalité des chercheurs dans le conflit localisé dans les zones périphériques. Néanmoins, le rôle de l'ICASEES au sein du gouvernement centrafricain a peut-être découragé certains participants de partager des informations sur les attaques s'ils se méfiaient du gouvernement. Deux enquêteurs non centrafricains, affiliés à des institutions universitaires américaines et européennes, ont également réalisé certains entretiens. Il est possible que certaines participants se soient senties plus à l’aise de partager des informations sur les attaques, en particulier l’identité des attaquants, au cours de ces entretiens, si elles estimaient que ces enquêteurs étaient moins susceptibles d’adopter une vision active du conflit ou de s’aligner sur des groupes armés particuliers. D’un autre côté, certains personnes interviewées se sont peut-être senties réticents à partager des informations sensibles avec des étrangers. La plupart des entretiens menés par les deux enquêteurs étrangers ont eu lieu avec du personnel expatrié d'ONG, ce qui, selon nous, aurait atténué une partie du risque de ce biais. Nous ne pouvons pas non plus garantir que les convictions personnelles, l’appartenance ethnique et la vision du conflit dans les trois préfectures des enquêteurs n’ont pas influencé la manière dont ils ont mené l’entretien. Cependant, nous n’avons pas identifié d’exemples aussi évidents lors de l’analyse des transcriptions des entretiens.

En raison de l’inégalité entre les sexes présente dans CAR(31), les informateurs clés étaient majoritairement des hommes. L’identification des informateurs clés a été difficile, et la participation des informatrices a été encore aggravée par la sous-représentation des femmes aux postes clés au sein du système de santé centrafricain. Cela représente une limite importante, car les perspectives et les impacts sur les agents de santé féminins sont probablement différents de ceux de leurs homologues masculins et les conclusions ici sont décrites en grande partie du point de vue des hommes. L’étude a utilisé une approche inductive de codage et d’analyse, ce qui comporte un risque de biais du chercheur, car une interprétation subjective peut influencer l’identification des thèmes et les résultats. Néanmoins, l’étendue et la cohérence des comptes garantissent la fiabilité des conclusions de l’étude.

## Conclusion

Les violences infligées aux soins de santé sont perpétrées en toute impunité et sont omniprésentes dans les régions de RCA touchées par le conflit. Le bilan psychologique et physique des attaques contre les prestataires et les communautés est profond, tandis que l'instabilité chronique et les défis opérationnels qui en découlent ont encore plus déstabilisé un système de santé qui souffre depuis longtemps d'un manque de ressources humaines en quantité et qualité et matérielles. La pauvreté et les déplacements généralisés, la médiocrité des communications et des infrastructures, la répartition inégale des ressources au niveau décentralisé, l’instabilité politique et la capacité limitée du gouvernement central à prévenir les attaques ou à atténuer leurs impacts exacerbent ces préjudices. Malgré les difficultés, les initiatives visant à soutenir les communautés qui subissent le plus gros de la violence, à renforcer les capacités et la formation des agents de santé et à développer de nouvelles stratégies de protection peuvent réduire les souffrances. Un effort concerté des parties prenantes locales et internationales est essentiel pour renforcer le système de santé en RCA, protéger ceux qui sont en première ligne et garantir à la population l’accès aux services vitaux.

## Déclarations

### Reconnaissances

### Nous exprimons notre plus profonde gratitude à l'équipe de ICASEES ainsi que de collecte de données, notamment aux enquêteurs, aux superviseurs, aux transcripteurs et au personnel de soutien, dont le dévouement a rendu cette étude possible. Des remerciements particuliers vont à M. Charles Daouili, directeur des ressources à l'ICASEES, et à M. Sébastien Selenguino, chef d'équipe de transcription, dont nous nous souvenons tous deux avec un profond respect suite à leur décès.

### Notre gratitude va également à l'International Medical Corps pour son soutien essentiel lors de la visite de cadrage initiale et des étapes ultérieures de l'étude, avec une mention particulière au Dr Christian Mulamba et au Dr Thierry Fikiri pour leurs contributions.

Nous remercions Sarah Woznick, qui a participé à la visite de cadrage et a contribué à façonner la conception initiale de l'étude. Prashasti Bhatnagar a contribué à l'élaboration du guide des informateurs clés.

### Nous reconnaissons les informations inestimables des membres de la RIAH, notamment Dr Karl Blanchet, Dre Larissa Fast, Stephani Rinaldi, Dre Rohini Haar et le lieutenant-général Louis Lillywhite, dont l'expertise a permis de situer notre travail dans le cadre d'une recherche plus large sur les soins de santé attaqués.

Nous sommes avant tout redevables aux participants à l’étude qui ont partagé leurs expériences avec nous. Leurs témoignages, reflétant le sacrifice, la résilience et la perte, ont joué un rôle déterminant dans l’élaboration de cette recherche. Nous espérons que ce travail rendra hommage à leurs histoires et contribuera à promouvoir la responsabilité et à réduire l’impact des attaques contre les soins de santé sur les survivants et les communautés.

### Approbation éthique et consentement à participer

L'étude a été approuvée par le Comité Ethique et Scientifique de l'Université de Bangui, République Centrafricaine (N°27/UB/FACSS/IPC/CES/021), par la Commission d'éthique Universitaire de l'Université de Genève (CUREG.202101.11). ), et par l'Institutionnel Review Board Office de l'Université Johns Hopkins (avis de détermination FWA #00000287). L’étude a reçu Quitus statistique du Comité Technique de Programmation des Activité Statistiques (CTPAS), République Centrafricaine (N°0377/2022/MEPC/DIRCAB/ICASEES/DMNER). Tous les participants ont donné leur consentement éclairé verbal ou écrit pour participer à l’étude.

Consentement pour la publication

Tous les auteurs ont donné leur consentement pour la publication. Au cours du processus de consentement éclairé, tous les participants ont approuvé l'utilisation des données à des fins scientifiques et la publication des résultats de recherche dans des revues ou des livres scientifiques, étant entendu que les données resteront anonymes et qu'aucune information d'identification ne sera incluse dans la publication.

Disponibilité des données et matériels

Les ensembles de données générés et/ou analysés au cours de la présente étude ne sont pas accessibles au public en raison de la nature sensible du contenu, mais sont disponibles auprès de l'auteur correspondant sur demande raisonnable.

Conflit d’intérêt

Aucun intérêt concurrent pour ce projet.

### Financement

Cette étude a été financée par le consortium Researching the Impacts of Attacks on Health Care soutenu par le ministère des Affaires étrangères du Royaume-Uni. Bureau du Commonwealth et du développement (UK FCDO).

### Contribution des auteurs

NK a contribué à la conception, au design, à l'acquisition des données, à l'analyse, à l'interprétation, à la rédaction et à la révision des travaux. JO a contribué à l'analyse, à l'interprétation, à la rédaction et à la révision. PS a contribué à la conception, au design, à l'acquisition et à la révision. BBA a contribué à l'acquisition et à la révision. AM a contribué à la conception, à l’acquisition, à l’analyse et à l’interprétation. OGB a contribué à l'acquisition et à l'examen. GM a contribué à l'acquisition et à l'examen. LR a contribué à la conception, au design, à l'interprétation, à la rédaction et à la révision.

### Abréviations

FOSA : Formation Sanitaire

ICASEES : Institut Centrafricain des Statistiques et des Etudes Economiques et Sociales

IMC : International Medical Corps

MINUSCA : et la Mission des Nations Unies en République Centrafricaine

MSP : Ministère de la Santé et de la Population

ONG : Organisation non-gouvernementale

RCA : République centrafricaine

SSA: WHO Surveillance System for Attacks on Health Care

## References

1. Political Geography Now. Central African Republic Control Map & Timeline - July 2021 2021 [cited 2023 Oct 10]. Available from: <https://www.polgeonow.com/2021/07/who-controls-the-central-african-republic-2021.html>.

2. United Nations Development Program. Human Development Report 2023/2024: Breaking the Gridlock.

3. United Nations Office for the Coordination of Humanitarian Affairs. Central African Republic Humanitarian Response Plan. 2023 2023 Jul 19.

4. United States Agency for International Development. Central African Republic – Complex Emergency Fact Sheet 1. 2023.

5. United Nations Development Program. Human Development Reports - Central African Republic: United Nations; 2024 [cited 2024 Dec 24]. Available from: <https://hdr.undp.org/data-center/specific-country-data#/countries/CAF>.

6. World Health Organization. Rapport Annuel, OMS en Republique Centralafrique. 2022.

7. United Nations Office for the Coordination of Humanitarian Affairs. 3W_CAR_Dec2023 (Who does What Where) République Centrafricaine : Présence Opérationnelle. 2024.

8. Safeguarding Health in Conflict. Violence On The Front Line: Attacks on Health Care in 2017. 2024.

9. Crawford K, Florez T, Rodriguez M, Cirado L, Read R, Haar R. “There is a fear that you will be attacked just for the act of working in health”: a survey of experiences of violence against healthcare in Colombia. Conflict and Health. 2023;17(1):51.

10. Barten DG, Tin D, Granholm F, Rusnak D, van Osch F, Ciottone G. Attacks on Ukrainian healthcare facilities during the first year of the full-scale Russian invasion of Ukraine. Confl Health. 2023;17(1):57.

11. Fouad FM, Sparrow A, Tarakji A, Alameddine M, El-Jardali F, Coutts AP, et al. Health workers and the weaponisation of health care in Syria: a preliminary inquiry for The Lancet-American University of Beirut Commission on Syria. Lancet. 2017;390(10111):2516-26.

12. Abbara A, Rayes D, Tappis H, Hamze M, Wais R, Alahmad H, et al. “Actually, the psychological wounds are more difficult than physical injuries:” a qualitative analysis of the impacts of attacks on health on the personal and professional lives of health workers in the Syrian conflict. Conflict and Health. 2023;17(1):48.

13. Neuman M. "No patients, no problems" Exposure to risk of medical personnel working in MSF projects in Yemen's governorate of Amran. J Humanit Assist. 2014.

14. Gesesew H, Kebede H, Berhe K, Fauk N, Ward P. Perilous medicine in Tigray: a systematic review. Confl Health. 2023;17(1):26.

15. International Rescue Committee. Joint Health Staff Survey: Protection of Health Care South Sudan. 2024 Oct.

16. International Rescue Committee. Joint Health Staff Survey Protection of Health Care In Northeast Nigeria. 2022 Oct.

17. Makali SL, Lembebu JC, Boroto R, Zalinga CC, Bugugu D, Lurhangire E, et al. Violence against health care workers in a crisis context: a mixed cross-sectional study in Eastern Democratic Republic of Congo. Confl Health. 2023;17(1):44.

18. Researching the Impact of Attacks on Healthcare. The University of Manchester; [cited 2024 Jan 30]. Available from: riah.manchester.ac.uk/.

19. QSR International Pty Ltd. NVivo (Version 14) 2023.

20. Safeguarding Health in Conflict. Ignoring Red Lines: Violence against Health Care in Conflict. 2024.

21. Physicians for Human Rights. Physicians for Human Rights’ Findings of Attacks on Health Care in Syria 2024 [cited 2024 Mar 18]. Available from: <https://syriamap.phr.org/#/en/findings>.

22. Mahmoud H, Abuzerr S. State of the health-care system in Gaza during the Israel-Hamas war. Lancet. 2023;402(10419):2294.

23. World Health Organization. Health Resources and Services Availability Monitoring System (HeRAMS) République Centrafricaine: Rapport de référence 2023 - Services des maladies non transmissibles et de santé mentale 2023.

24. Al Waziza R, Sheikh R, Ahmed I, Al-Masbhi G, Dureab F. Analyzing Yemen's health system at the governorate level amid the ongoing conflict: a case of Al Hodeida governorate. Discov Health Syst. 2023;2(1):15.

25. Ministry of Health and Population - Central African Republic, WHO. Enquête Nationale sur Les Prestations des Services de Santé (SARA/HeRAMS). 2019.

26. Médecins Sans Frontières. Unprotected: Report on violence and lack of protection of civilians in CAR 2019.

27. World Health Organization. Health Resources and Services Availability Monitoring System (HeRAMS). 2019.

28. World Health Organization. Rapport De Situation No 1 Rougeole 2021. 2024.

29. Ahmat A, Okoroafor SC, Kazanga I, Asamani JA, Millogo JJS, Illou MMA, et al. The health workforce status in the WHO African Region: findings of a cross-sectional study. BMJ Glob Health. 2022;7(Suppl 1).

30. World Health Organization. Health workforce requirements for universal health coverage and the sustainable development goals.(human resources for health observer, 17). 2016.

31. United Nations Development Program. Human Development Report 2021-22. UNDP (United Nations Development Programme). 2022.

32. Elnakib S, Elaraby S, Othman F, BaSaleem H, Abdulghani AlShawafi NA, Saleh Al-Gawfi IA, et al. Providing care under extreme adversity: The impact of the Yemen conflict on the personal and professional lives of health workers. Soc Sci Med. 2021;272:113751.

33. Singh NS, Redman B, Broussard G, DeCamp M, Rayes D, Ho LS, et al. 'We will never give up': a qualitative study of ethical challenges Syrian health workers face in situations of extreme violence. Disasters. 2022;46(2):301-28.

34. Carter W, Haver K. Humanitarian Access Negotiations with Non-State Armed Groups. Secure Access in Volatile Environments (SAVE); 2016 Oct.

35. Barbelet V, Soignet KME, Yidong MC. Community Engagement With Armed Actors in the Central African Republic. Humanitarian Policy Group; 2023 Oct.

36. La RCA opte pour des réformes profondes du secteur santé [press release]. 2023 Aug 23,.

37. The World Bank. Implementation Completion and Results Report, CAR Health System Support and Strengthening (SENI) Project. 2023.

38. Rubenstein L. Perilous Medicine: The Struggle to Protect Health Care from the Violence of War: Columbia University Press; 2021.

39. Bax P. Russia's Influence in the Central African Republic 2021 [cited 2024 Mar 18]. Available from: <https://www.crisisgroup.org/africa/central-africa/central-african-republic/russias-influence-central-african-republic>.

40. Lechner J, Ingasso V. Wagner Woes and a Rebel Crackdown: A Briefing on the Central African Republic's Shifting Conflict: The New Humanitarian; 2023 Sept 7 [cited 2024 Feb 12]. Available from: <https://www.thenewhumanitarian.org/analysis/2023/09/07/wagner-woes-and-rebel-crackdown-briefing-central-african-republics-shifting>.

41. Yongo JS, T., McAllister, E. Felix, B., Bell, A. . France suspends aid, military support for Central African Republic 2021 [cited 2024 Mar 27]. Available from: <https://www.reuters.com/world/africa/france-suspends-aid-military-support-central-african-republic-2021-06-08/>.

42. ECORYS. Evaluation de la coopération de l’Union européenne avec la République Centrafricaine

Rapport final. Commission Européenne; 2021.

43. Jarrett P, Checchi F. Aid to the Central African Republic falls while death rates rise. Lancet. 2023;402(10415):1830-1.

44. The World Bank. Net Official Development Assistance and Official Aid Received (current USD) 2021 [cited 2024 Feb 15]. Available from: <https://data.worldbank.org/indicator/DT.ODA.ALLD.CD?locations=CF&most_recent_value_desc=true>.

45. United Nations Office for the Coordination of Humanitarian Affairs. Central African Republic: Situation Report, 25 Mar 2024. 2024.

46. World Health Organization. Surveillance System For Attacks on Healthcare (SSA) 2024 [cited 2024 Mar 27]. Available from: <https://extranet.who.int/ssa/LeftMenu/Index.aspx>.

47. Haar R, Sirkin, S. Strengthening Data to Protect Healthcare in Conflict Zones. International Peace Institute; 2022.

48. International Criminal Court. The Prosecutor of the International Criminal Court, Karim A.A. Khan KC, announces conclusion of the investigation phase in the Situation in the Central African Republic 2022 Dec 16 [cited 2023 Dec 27]. Available from: <https://www.icc-cpi.int/news/prosecutor-international-criminal-court-karim-aa-khan-kc-announces-conclusion-investigation-0>.

49. Ndiyun RK. The Special Criminal Court and the challenge of criminal accountability in the Central African Republic. SN Social Sciences. 2023;3(9):147.

50. Human Rights Watch. Central African Republic: First Trial at the Special Criminal Court 2022 Apr 12 [cited 2024 Dec 27]. Available from: <https://www.hrw.org/news/2022/04/12/central-african-republic-first-trial-special-criminal-court>.

51. Mudge L. Rearrest Sparks Hope in Central African Republic: Human Rights Watch; 2023 [cited 2024 Mar 16]. Available from: <https://www.hrw.org/news/2023/09/08/rearrest-sparks-hope-central-african-republic>.

52. Chandini MA, Van den Bergh R, Agbor Junior AA, Willliam F, Obi AMM, Ngeha NC, et al. "It is because of the love for the job that we are still here": Mental health and psychosocial support among health care workers affected by attacks in the Northwest and Southwest regions of Cameroon. PLOS Glob Public Health. 2023;3(11):e0002422.

1. La définition de la mortalité hospitalière prend en compte toutes les personnes décédées qui ont été amenées ou sont décédées dans l'établissement de santé et dont le décès a été confirmé par un agent de santé. [↑](#footnote-ref-1)
